# Wnt signalling maintains self-renewal of human hepatoblasts without blocking their differentiation

**DOI:** 10.1242/dev.205026

**Published:** 2025-11-20

**Authors:** Ekaterini D. Zacharis, Carola M. Morell, Rute A. Tomaz, Arash Shahsavari, Charlotte Grey-Wilson, Maïa Pesic, Vasileios Galanakis, Eleanor C. Williams, Irina Mohorianu, Irene Talon, Ludovic Vallier

**Affiliations:** ^1^ Wellcome-MRC Cambridge Stem Cell Institute, University of Cambridge, Cambridge CB2 0AW, UK; ^2^ Department of Surgery, University of Cambridge, Cambridge CB2 0QQ, UK; ^3^ Max Planck Institute for Molecular Genetics, Berlin 14195, Germany; ^4^ Centre for Regenerative Therapies (BCRT), Berlin Institute of Health (BIH), Charité - Universitätsmedizin, Berlin 13353, Germany; ^5^ Liver Unit, Department of Medicine, Cambridge NIHR Biomedical Research Centre, Cambridge University Hospitals NHS Foundation Trust, Cambridge CB2 0QQ, UK

**Keywords:** Hepatoblast, Organoid, Liver, Hepatocyte, Cholangiocyte

## Abstract

Hepatoblasts play a key role in liver organogenesis by differentiating into hepatocytes and cholangiocytes, the main functional cell types of the liver. Mouse studies have demonstrated an association of Wnt signalling with proliferation and differentiation of hepatoblasts. However, the exact function of this pathway in hepatic development has not been fully uncovered, especially in human. Here, we use hepatoblast organoids derived from human foetal livers to investigate the importance of Wnt signalling in self-renewal and cell fate decisions during liver development. We first showed that Wnt plays a key role in hepatoblast self-renewal capacity *in vitro* by maintaining their proliferative state. However, Wnt was not sufficient to block differentiation of hepatoblast organoids into hepatocytes or cholangiocytes, suggesting that other factors are necessary to maintain hepatoblast bipotency. Finally, single-cell transcriptomic analyses revealed that Wnt signalling activity correlates with hepatoblast proliferation in the human foetal liver, suggesting that the role for Wnt could be conserved *in vivo*. Taken together, our results support a model in which Wnt signalling acts to preserve the proliferative capacity of hepatoblasts without being sufficient to maintain their bipotent state.

## Teaser

**Summary:** Analysis of human hepatoblast organoids reveals that Wnt signalling plays a key role in hepatoblast self-renewal by maintaining proliferation without altering their identity or differentiation into hepatocytes or cholangiocytes.

## INTRODUCTION

The development of the liver proper starts with the formation of the liver bud in the posterior part of the foregut. This region comprises hepatic endoderm cells that differentiate into hepatoblasts, representing the earliest stem cells of the liver. Hepatoblasts display a high proliferative capacity, necessary for the expansion of the liver parenchyma. They are characterised by a defined transcriptional network comprising TBX3, GATA4/6 and ONECUT transcription factors and the expression of specific markers such as AFP (Zorn et al., 2009; Gordillo et al., 2015; Ober and Lemaigre, 2018; Lotto et al., 2023). Following proliferation and migration into the surrounding mesenchyme, hepatoblasts differentiate into hepatocytes or cholangiocytes, depending on their location within the developing liver. Hepatoblasts close to the periportal mesenchyme are exposed to TGFβ and Notch signalling and can become cholangiocytes through formation of the ductal plate around blood vessels (Kaylan et al., 2016). Hepatoblasts that receive signals, such as oncostatin M (OSM) and hepatocyte growth factor (HGF), from surrounding haematopoietic progenitors, differentiate into foetal hepatocytes (Kamiya, 1999; Michalopoulos et al., 2003).

Importantly, genetic and lineage-tracing studies in animal models have shown that Wnt signalling is necessary for hepatoblast self-renewal *in vivo*. More precisely, at early stages of hepatic specification, absence of Wnt signalling allows the expression of Hhex in the liver bud (McLin et al., 2007). However, in later stages of development, bipotent mouse hepatoblasts express the Wnt target gene *Lgr5* during early hepatogenesis (Prior et al., 2019), and the Wnt pathway sustains hepatoblasts during later stages of organogenesis and in disease (Suksaweang et al., 2004; Perugorria et al., 2018). In addition, Wnt influences the differentiation of hepatoblasts towards the hepatic or biliary lineages. Of particular interest, knockout mice for *Ctnnb1* (β-catenin) display fewer hepatocytes with decreased functionality (Tan et al., 2008). Loss of APC, a protein complex that targets Ctnnb1 for destruction, drives hepatoblast differentiation toward the biliary lineage thereby affecting hepatocyte specification (Decaens et al., 2007). Thus, Wnt seems to be necessary for both self-renewal and differentiation of hepatoblasts in liver development. Further studies are therefore necessary to understand the mechanisms by which Wnt fulfils these contradictory functions. However, this question is particularly challenging to tackle *in vivo* due to the complexity of the foetal liver, the transitory nature of hepatoblasts, which progressively disappear during organogenesis, and the challenge in accessing primary tissues in human.

To address this question, we investigated the roles of Wnt signalling pathway in the human foetal liver using hepatoblasts grown *in vitro* as organoids. We recently established a culture system for growing and differentiating human hepatoblast organoids (HBOs), which closely resemble their *in vivo* counterpart (Wesley et al., 2022). HBOs are composed of a homogeneous population of hepatoblasts. They can self-renew while displaying a transcriptomic profile characteristic of hepatoblasts and they maintain the capacity to differentiate into hepatocytes and biliary progenitors (Wesley et al., 2022). Taking advantage of this *in vitro* model, we first confirmed the importance of the Wnt pathway in hepatoblast self-renewal and cell fate choice. We then demonstrated that hepatoblast identity and differentiation capacity are both independent of Wnt regulation. Instead, we showed that Wnt signalling is necessary for the proliferation and survival of HBOs. This activity is achieved through the regulation of cell cycle inhibitors and apoptosis-related genes. Finally, we observed a causal link between Wnt signalling activation and proliferation during development of human foetal liver using single-cell transcriptomic analyses. Together, our results suggest that Wnt is not sufficient to block differentiation of hepatoblast but is necessary to maintain their self-renewal. Such findings explain in part the contradictory function of Wnt during liver development and could be broadly applicable to a number of stem cells in adult tissues.

## RESULTS

### Human hepatoblasts grown as organoids display an active Wnt signalling pathway that is not essential for their short-term self-renewal

We previously developed culture conditions to grow human hepatoblasts derived from foetal livers aged 5-10 post-conceptional weeks (pcw) as organoids (Wesley et al., 2022) (Fig. S1A). The resulting HBOs strongly resemble their primary counterparts, while maintaining the capacity to differentiate into both hepatocytes and cholangiocytes. Interestingly, these culture conditions are based on media containing WNT3A (WNT) and R-spondin 1 (RSPO), suggesting that HBOs could represent a relevant model to study the role of Wnt signalling pathway during early human liver development *in vitro*. To test this hypothesis, we first confirmed that HBOs expressed hepatoblast markers, including ALB and AFP (Fig. S1B,C) (Petkov et al., 2004). Moreover, HBOs displayed effectors and target genes of Wnt pathway activity, including active β-catenin (ABC) and AXIN2 (Fig. S1B,C). Given our ability to culture HBOs, we then investigated the importance of Wnt in self-renewal of HBOs. We first grew HBOs for 7 days in the absence of WNT3A (−WNT +RSPO), RSPO (−RSPO +WNT) or both (−RSPO −WNT). To ensure that endogenous Wnt factors did not influence our loss-of-function experimental condition, HBOs were also grown in the absence of WNT3A combined with the presence of RSPO and the Wnt inhibitor DKK1 (−WNT +RSPO +DKK1). Finally, HBOs were grown in the presence of the WNT agonist CHIR99021 (CHIR).

**Fig. 1.**
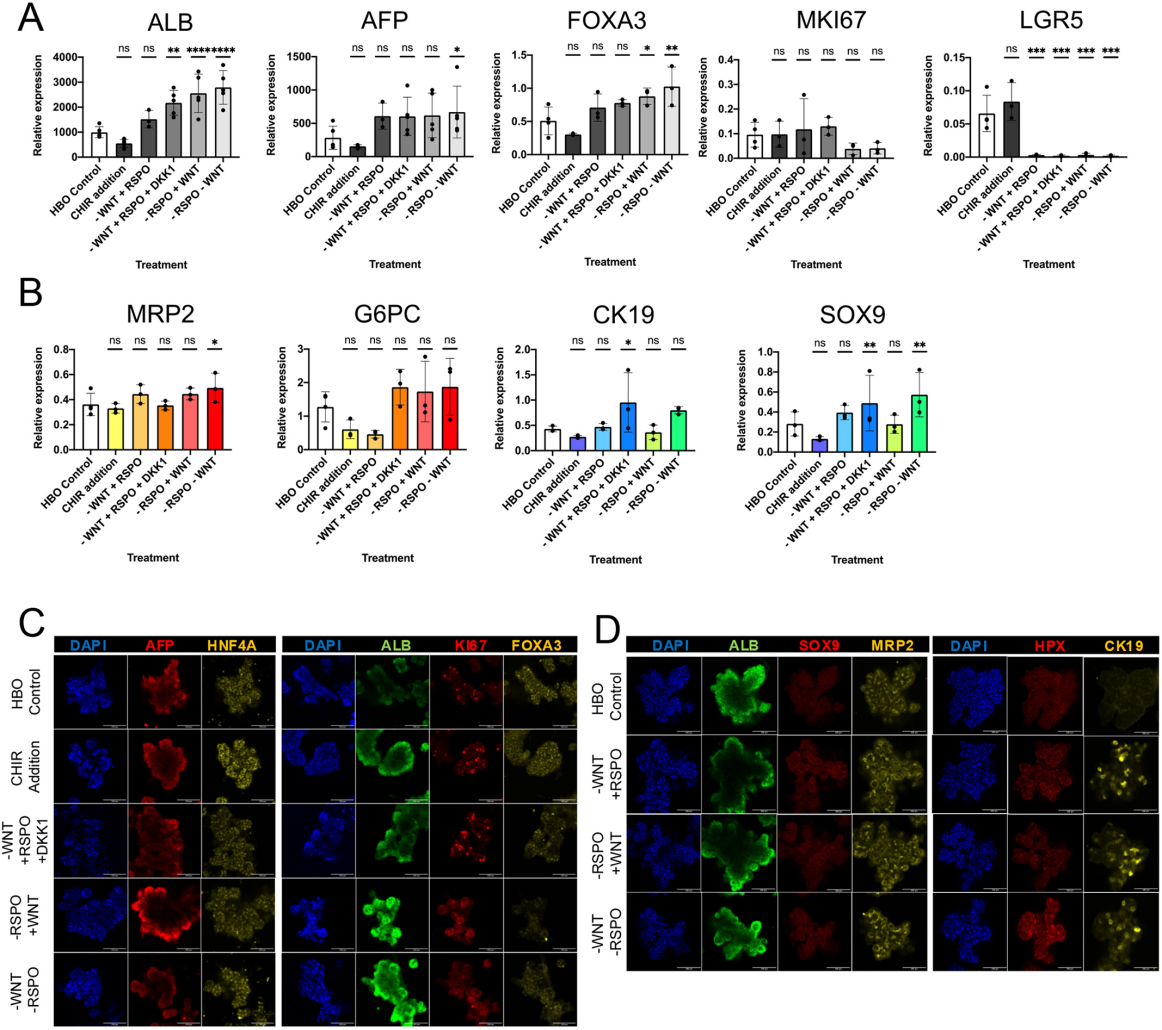
**Absence of Wnt signalling does not induce differentiation of HBOs.** (A,B) qPCR analyses showing the expression of the denoted genes, including hepatoblast markers (A) and mature hepatic and biliary genes (B) in HBOs grown in culture conditions with enhanced or reduced Wnt signalling. HBOs grown in maintenance conditions were used as a control. (C) Immunofluorescence staining showing the expression of the denoted proteins in HBOs grown in maintenance conditions (HBO Control), in the presence of CHIR and in the absence of WNT3A, RSPO or both combined. (D) Immunofluorescence staining showing the expression of the foetal hepatocyte markers (HPX, MRP2) and biliary markers (SOX9, CK19) in HBOs grown in the different culture conditions. qPCR data are shown as mean±s.d. in three to five independent biological replicates (*n*=3-5), where *P<0.05, **P<0.01, ***P<0.001, ****P<0.0001 (one-way ANOVA). ns, not significant. Images are representative of 3 samples.

Additional Wnt activation by CHIR had little effect on hepatoblast markers or differentiation markers, suggesting that control conditions are already optimal for Wnt signalling activity (Fig. 1A-C). Withdrawing WNT and/or RSPO, or adding DKK1, abolished the expression of *LGR5*, confirming inhibition of the Wnt pathway (Fig. 1A). However, markers for hepatoblasts or for differentiation into hepatocytes were consistently not affected by Wnt inhibition, with the exception of *ALB*, which was significantly increased (Fig. 1A). We did not detect changes in hepatoblast (*AFP* and *FOXA3*), proliferation (*MKI67*) and hepatocyte [*MRP2* (*ABCC2*) and *G6PC*] markers (Fig. 1A,B). These observations were confirmed at the protein level using immunostaining (Fig. 1C,D) and suggest that Wnt withdrawal does not drive hepatoblasts towards the hepatocyte fate under the tested conditions.

At the transcriptomic level, the early biliary marker *SOX9* was significantly upregulated in the absence of WNT and upon Wnt signalling inhibition by DKK1 (Fig. 1B). In addition, the biliary marker *CK19* (KRT19) was significantly upregulated upon Wnt inhibition by DKK1 (Fig. 1B). However, hepatoblast and hepatocyte markers did not undergo downregulation under these conditions (Fig. 1A), indicating that these cells still maintain their hepatoblast identity. At the protein level, absence of Wnt was associated with the emergence of cells with high levels of CK19 protein (Fig. 1D). Of note, these cells continued to express *ALB*, suggesting that they could be equivalent to the early ductal plate progenitors observed *in vivo* during the first stage of the intrahepatic biliary tree development (Carpentier et al., 2011). To confirm this hypothesis, we performed immunostaining analyses on human foetal tissue slides and observed the presence of similar biphenotypic cells in the ductal plate surrounding blood vessels (Fig. S2). Taken together, these results suggest that Wnt signalling is not necessary for short-term self-renewal of HBOs *in vitro*.

### Long-term absence of Wnt limits self-renewal capacity of hepatoblasts

**Fig. 2.**
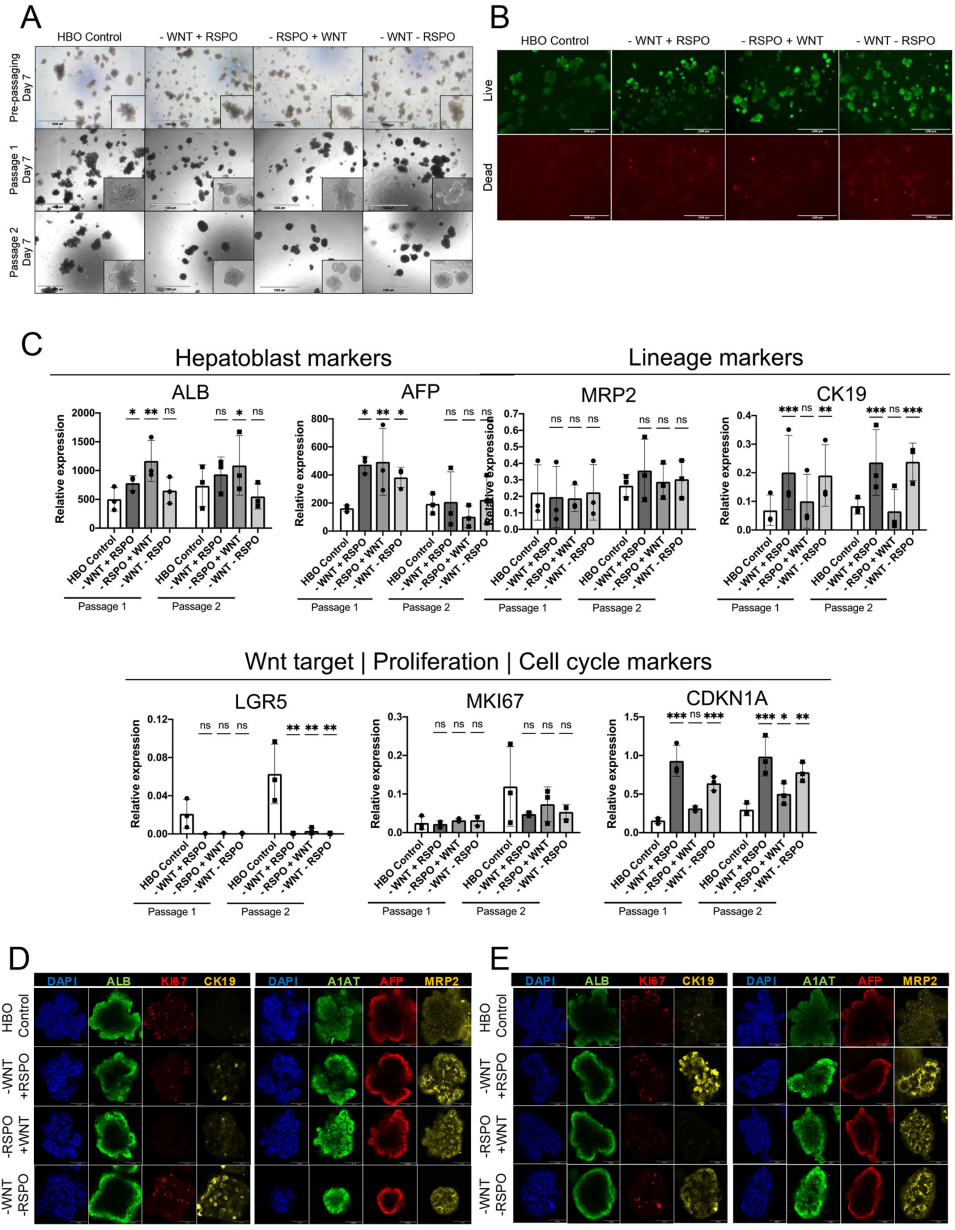
**Wnt signalling is necessary for HBO self-renewal.** (A) Brightfield images showing HBOs grown for 7 days, 14 days (passage 1) and 21 days (passage 2) in the presence or absence of WNT3A and/or RSPO. Insets show a zoomed in picture of a representative organoid. Scale bars: 1000 µm. (B) Assay for live (green) and dead (red) cells in HBOs grown in the absence of Wnt signalling for 14 days. Scale bars: 1000 µm. (C) qPCR analyses for the expression of denoted genes in HBOs grown for 14 or 21 days (passage 1 and 2, respectively) in the presence or in the absence of Wnt signalling molecules. (D,E) Immunofluorescence staining for KI67, ALB, AFP, A1AT, MRP2 and CK19 on HBOs grown for 14 days (D) or 21 days (E) in the presence or absence of WNT3A and/or RSPO. Scale bars: 100 µm. qPCR data are shown as mean±s.d. in three independent biological replicates (*n*=3), where *P<0.05, **P<0.01, ***P<0.001 (two-way ANOVA). ns, not significant. Images are representative of 3 samples.

Developmental studies have shown that Wnt signalling is necessary for liver development (Suksaweang et al., 2004; Lade and Monga, 2011; Perugorria et al., 2018; Prior et al., 2019). Thus, we decided to investigate the importance of Wnt signalling in long-term self-renewal of HBOs. For that, HBOs were grown in the absence of WNT3A (−WNT +RSPO), RSPO (−RSPO +WNT) or both (−WNT −RSPO), for 14 and for 21 days and were passaged once or twice during this time, respectively. Expansion in reduced WNT3A and RSPO led to morphological changes in HBOs with decreased size, lack of budding and a more circular shape (Fig. 2A). Eventually, HBOs grown in the absence of Wnt factors died and could not be split for a third time. This observation was confirmed by staining of live and dead cells (Fig. 2B), showing an increase in cell death after the first passage in culture conditions lacking Wnt factors. Of note, this effect was aggravated by the combined absence of WNT3A and RSPO, demonstrating the synergistic effect of these factors on HBOs self-renewal.

We then investigated whether long-term absence of Wnt signalling factors induces the expression of differentiation makers. Immunostaining and gene expression analyses confirmed that Wnt is necessary for *LGR5* expression but not for the expression of the hepatoblast markers *AFP* and *ALB*, which suggests the absence of differentiation toward hepatocytes or cholangiocytes (Fig. 2C,D). Accordingly, a lack of Wnt factors had little effect on the expression of *CK19* and *MRP2* at the transcript level (Fig. 2C). However, CK19 protein seemed to increase in a subset of hepatoblasts that remained positive for AFP or ALB (Fig. 2D,E). Similarly, MRP2 protein also seemed to increase in HBOs upon WNT3A/RSPO depletion, suggesting that Wnt signalling could affect the stability of specific proteins (Fig. 2D,E). Of note, we also analysed markers for cell cycle progression and regulation to determine whether the absence of Wnt could affect proliferation (Fig. 2C-E; Fig. S3). These analyses revealed that absence of Wnt factors had little effect on *CDK1* or *MKI67* expression, while *CDKN1A* (p21), often associated with quiescence or senescence, and *ATF5*, linked with cell cycle arrest in the liver (Gho et al., 2008), were upregulated (Fig. S3). These observations suggest that Wnt signalling does not block differentiation of hepatoblasts. However, it plays a key role in hepatoblast self-renewal by promoting cell survival and potentially limiting the induction of senescence and quiescence markers.

### The presence of Wnt does not block differentiation of hepatoblasts into foetal hepatocytes or cholangiocytes

**Fig. 3.**
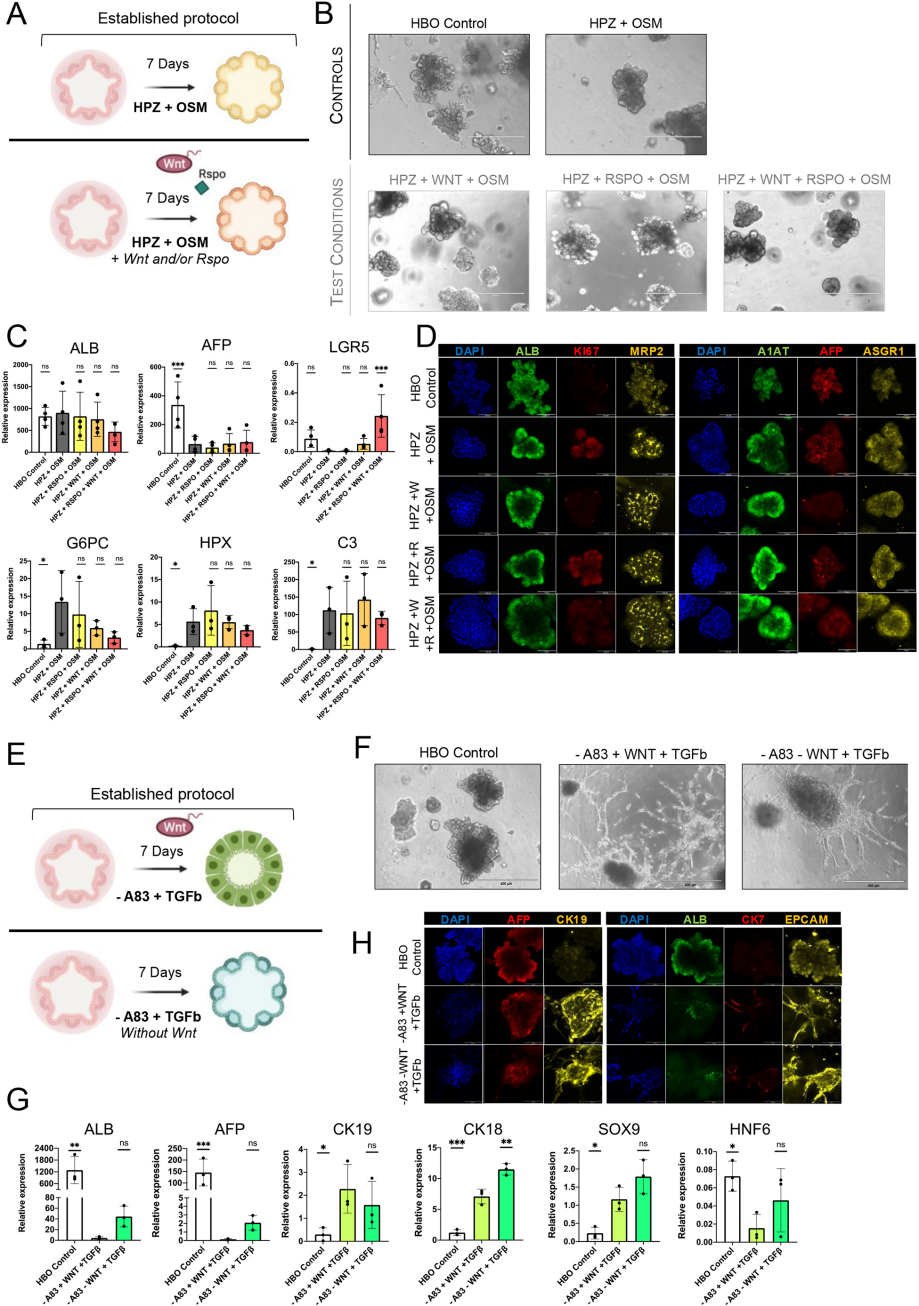
**Addition of WNT3A and RSPO does not block HBO differentiation into foetal hepatocytes and biliary cells.** (A) Schematic showing the method for differentiating HBOs into foetal hepatocytes in the presence or absence of Wnt signalling. (B) Brightfield images of HBOs grown in culture conditions supporting their self-renewal (HBO control), inducing their differentiation into foetal hepatocytes (HPZ+OSM) and in the same conditions with the presence of WNT3A and/or RSPO (HPZ+WNT+OSM; HPZ+RSPO+OSM; HPZ+WNT+RSPO+OSM). Scale bars: 400 μm. (C) qPCR analyses showing the expression of markers for HBOs (*ALB*, *AFP*), the Wnt target gene *LGR5* and foetal hepatocytes (*G6PC*, *HPX*, and *C3*). HBOs differentiated into foetal hepatocytes were used as control. (D) Immunofluorescence staining showing the expression of the denoted proteins in HBOs grown in the different conditions described above. Scale bars: 100 μm. (E) Schematic showing the method for differentiating HBOs into biliary cells in the presence or absence of WNT3A. (F) Brightfield images of HBOs grown in culture conditions supporting their self-renewal (control) and in culture conditions inducing their differentiation into biliary cells (−A83+WNT+TGFb) and in the same conditions without WNT3A (−A83−WNT+TGFb). Scale bars: 400 µm. (G) qPCR analyses showing the expression of the denoted genes in HBO differentiated into biliary cells in the presence or absence of WNT3A. Relative expression of hepatoblast markers *ALB*, *AFP* and mature biliary markers *CK19*, *CK18*, *SOX9* and *HNF6* on HBOs and biliary differentiation controls were compared to WNT3A removal from the latter. (H) Immunofluorescence staining showing the expression of the denoted proteins in undifferentiated HBOs and in HBOs grown in culture conditions inducing biliary differentiation with and without WNT3A. Scale bars: 100 µm. qPCR data are shown as mean±s.d. in three to four independent biological replicates (*n*=3-4), where *P<0.05, **P<0.01, ***P<0.001 (two-way ANOVA). ns, not significant. Images are representative of 3 samples.

To strengthen our observations, we decided to investigate the capacity of WNT3A and RSPO to block differentiation. HBOs were induced to differentiate into foetal hepatocytes with Hepatozyme medium supplemented with OSM (HPZ+OSM), as described previously (Wesley et al., 2022), in the absence and presence of WNT3A and/or RSPO (Fig. 3A). Addition of WNT3A, RSPO or both did not alter the morphology of differentiating HBOs (Fig. 3B), or the induction of foetal hepatocyte markers (Fig. 3C). Indeed, WNT3A and/or RSPO were not sufficient to maintain the expression of the hepatoblast marker *AFP* or to block the increase in hepatocyte markers such as *ALB*, *G6PC*, *HPX* and *C3* (Fig. 3C). Of note, *G6PC* is also a marker of liver zonation, which could be controlled by the Wnt signalling gradient (Jonker et al., 1996) and thus variation in the expression of this gene might reflect changes in both differentiation and functional specialisation. Finally, these observations were confirmed at the protein level for AFP, ALB, A1AT (SERPINA1), KI67 (MKI67), MRP2 and ASGR1 (Fig. 3D). Collectively, these results show that Wnt signalling is not sufficient to block the differentiation of hepatoblasts into hepatocytes.

Next, HBOs were differentiated into biliary cells by supplementing HBO culture conditions with TGFβ (Wesley et al., 2022) (Fig. 3E), a growth factor known to drive specification of early cholangiocytes *in vivo* (Clotman et al., 2005). Similar to hepatocyte differentiation, absence or presence of WNT3A had little effect on the organoid morphology (Fig. 3F) and the induction of biliary markers associated with cholangiocyte specification [*KRT18*, *KRT19*, *SOX9* and *HNF6* (*ONECUT1*)] (Fig. 3G). In addition, *AFP* and *ALB* were also downregulated, confirming that Wnt signalling is not sufficient to block differentiation of HBOs toward biliary cells (Fig. 3G). Immunofluorescence confirmed that HBOs differentiated into cholangiocytes with or without WNT3A lack AFP and ALB expression while inducing biliary markers [CK19, CK7 (KRT7) and EPCAM] (Fig. 3H). Taken together, these results show that Wnt signalling is not sufficient to block hepatoblast differentiation towards the biliary or hepatocytic lineages.

### Wnt signalling does not block commitment during hepatocyte differentiation but increases proliferative capacity

**Fig. 4.**
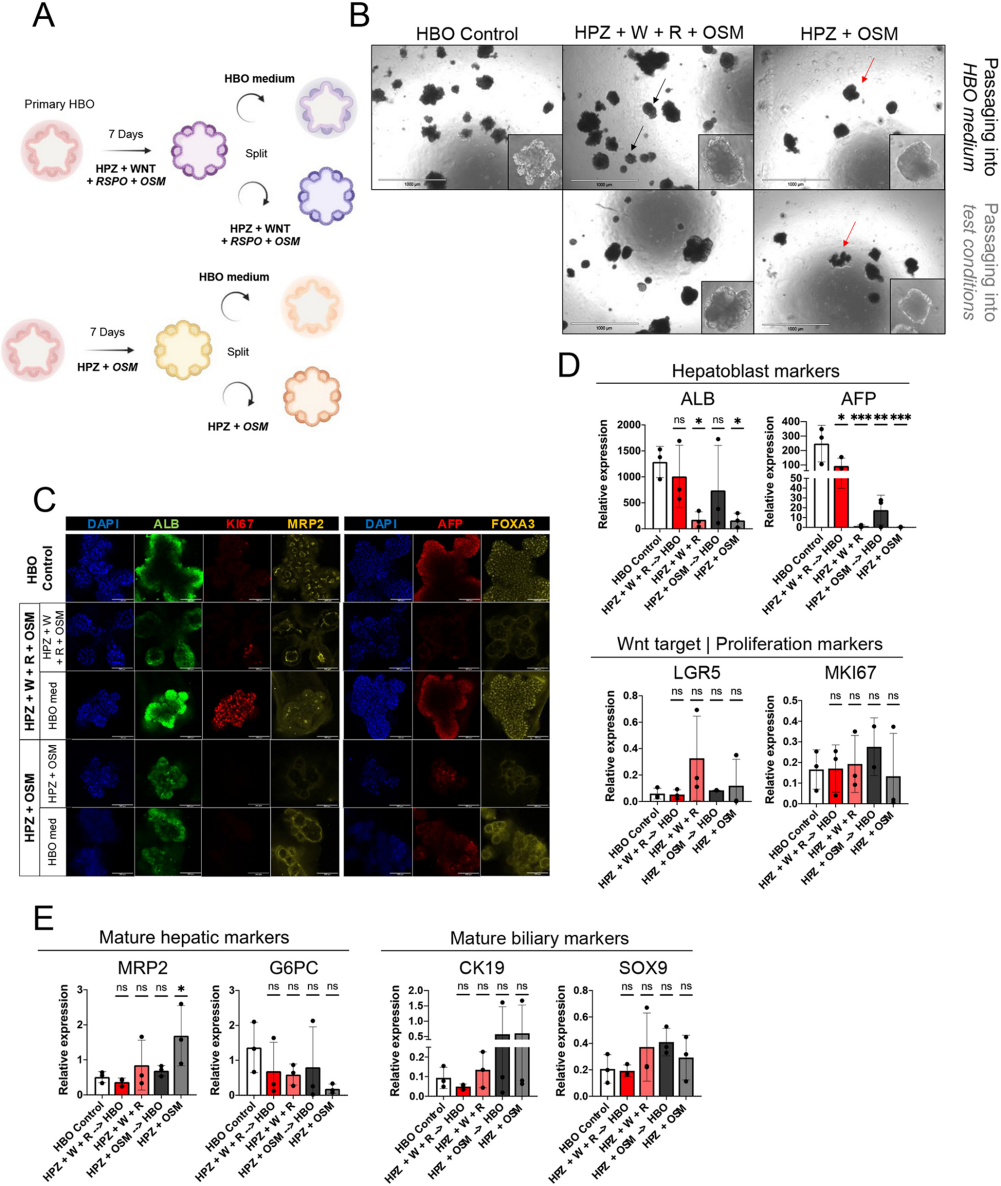
**Wnt signalling limits the commitment of HBOs toward the hepatocyte pathway.** (A) Schematic of the experimental design. Hepatic organoids were generated ±WNT3A and RSPO and then split in culture conditions driving differentiation or supporting HBO self-renewal. (B) Brightfield images of HBOs grown in the denoted culture conditions; organoids in hepatic differentiation media with WNT3A and RSPO appear healthier (black arrows) than those grown without (red arrows). R, RSPO; W, WBT3A. Insets show a zoomed in picture of a representative organoid. Scale bars: 1000 µm. (C) Immunofluorescence staining for the expression of ALB, AFP, FOXA3, MRP2 and the proliferation marker KI67 on HBOs and hepatic organoids in respective conditions. Scale bars: 100 µm. (D,E) qPCR analyses for the expression of hepatoblast, Wnt target and proliferation markers (D), and hepatic and biliary lineage markers (E) on HBOs treated in hepatic differentiation medium only (HPZ+OSM) or with WNT3A and RSPO added (HPZ+WNT+RSPO) split into their respective media or HBO maintenance medium. qPCR data are shown as mean±s.d. in three independent biological replicates (*n*=3), where *P<0.05, **P<0.01, ***P<0.001 (two-way ANOVA). ns, not significant. Images are representative of 3 samples.

Having demonstrated that Wnt has little impact on biliary or hepatocytic differentiation, we decided to investigate whether it could block the commitment of hepatoblasts toward the hepatocyte lineage. To test this hypothesis, HBOs were differentiated into foetal hepatocytes in the presence or absence of WNT3A and RSPO. The resulting hepatic organoids were then split into their respective differentiation media or transferred back into culture conditions supporting HBO self-renewal for 9 days (Fig. 4A). In the absence of Wnt factors (−WNT3A, −RSPO), differentiated HBOs adopted morphology indicative of lack of self-renewal and cell death, including absence of budding (Fig. 4B, red arrows). These organoids also lost the expression of hepatoblast markers, including *ALB* and *AFP* (Fig. 4D), suggesting that their phenotype is not stable *in vitro* and that they could dedifferentiate. Importantly, the addition of Wnt factors after differentiation was not sufficient to recover HBOs or hepatocyte markers, suggesting that Wnt signalling is not enough to block this dedifferentiation process. However, HBOs differentiated in the presence of WNT3A and RSPO could grow in size (Fig. 4B, black arrows). These observations were confirmed by immunostaining and gene expression analyses, showing that foetal hepatocyte organoids generated in the presence of Wnt signalling were able to re-express hepatoblast markers such as AFP, FOXA3, and the proliferation marker KI67 (Fig. 4C-D), while losing the expression of differentiation markers such as *MRP2* and *G6PC* (Fig. 4E). Of note, the cholangiocyte markers *CK19* and *SOX9* were not induced in any of the conditions described above (Fig. 4E). Taken together, these data show that Wnt signalling is not sufficient to block differentiation of HBOs toward the hepatocyte lineage. However, the presence of WNT3A and RSPO preserves the proliferation capacity of differentiating cells while maintaining their ability to re-express hepatoblast markers.

### Wnt signalling is not necessary to maintain the transcriptional profile characterising hepatoblasts

**Fig. 5.**
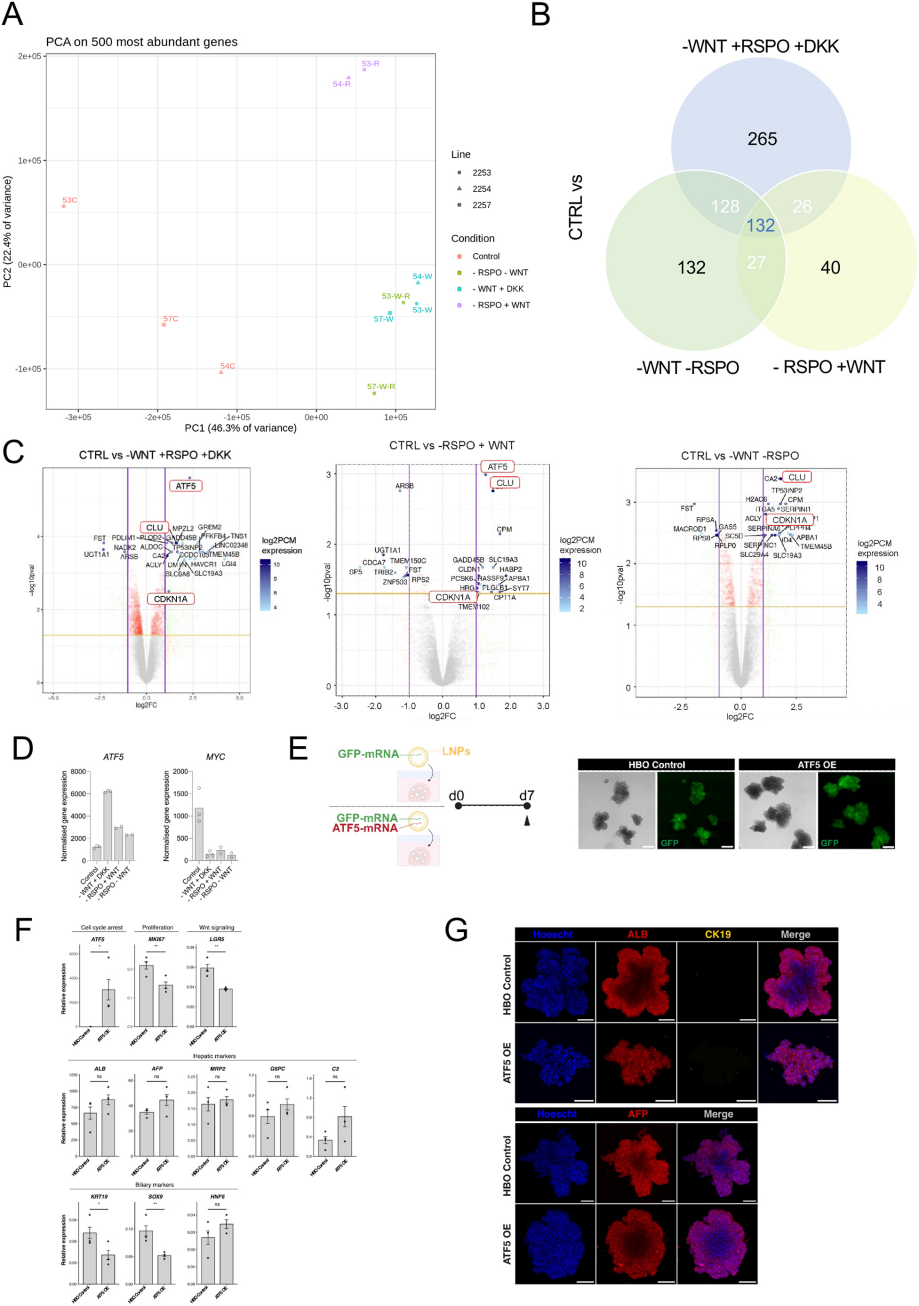
**Expression profile of HBOs grown in the presence of the absence of Wnt signalling.** (A) PCA showing the impact of WNT3A and/or RSPO on the gene expression profile of HBOs. (B) Venn diagram indicating uniquely and shared DEGs amongst the different comparisons (log2 fold change≥1). (C) Volcano plots showing the top 20-25 genes differentially expressed in HBOs grown in the reduced WNT3A/RSPO culture conditions against the control. Red boxes indicate genes of biological interest and those that are common among comparisons. Each dot represents a separate gene; plotted is Log2FC (fold change) against the −Log10 of the adjusted *P*-value for each comparison and gene investigated. (D) Gene expression levels, determined by RNA-seq analysis for *ATF5* and *MYC*. Individual data points from *n*=2-3 biologically independent samples are shown. The bar indicates the mean expression value. (E) Left: Schematic showing ATF5 OE in HBOs using LNPs. Right: Brightfield and GFP images of HBOs treated with GFP-mRNA LNPs (control) and ATF5-GFP-mRNA LNPs (ATF5 OE) 7 days after transfection. Scale bars: 250 µm. d, day. Arrowhead in schematic indicates the day of collection. (F) qPCR analyses for the expression of the denoted genes in HBOs transfected with HBO control and ATF5 OE after 7 days. qPCR data are shown as mean±s.e.m. in three independent technical replicates (*n*=3); *P<0.05, **P<0.01 (two-tailed *t*-test). (G) Immunofluorescence staining for ALB, CK19 and AFP on HBO control and ATF5 OE after 7 days. Scale bars: 100 µm. ns, not significant. Images are representative of 3 samples.

To further understand the role of Wnt signalling in HBOs with increased resolution, we carried out RNA sequencing (RNA-seq) and compared the transcription profile of HBOs grown in control conditions (with WNT3A and with RSPO) versus HBOs grown without WNT3A and RSPO (−RSPO −WNT), without WNT3A and with DKK1 (−WNT +RSPO +DKK1) and without RSPO (−RSPO +WNT). For convenient access to the RNA-seq data, we created a searchable tool to explore the data (https://bioinf.stemcells.cam.ac.uk/shiny/vallier/WntHepatoblast/) (Moutsopoulos et al., 2023). Principal component analysis (PCA) on the 500 most abundant genes (Fig. 5A) revealed that HBOs grown in control conditions (with WNT3A and RSPO) show different transcriptional profile compared to HBOs grown in the absence of WNT3A with DKK1, RSPO or without both WNT3A and RSPO (Fig. 5A, PC1). Interestingly, we also observed that HBOs react differently to the absence of RSPO versus WNT3A (Fig. 5A, PC2), suggesting that each factor could have overlapping but also separate activity. Next, we performed differential expression gene (DEG) analyses and observed that a total of 747 genes significantly changed their expression upon Wnt signalling modulation in the different culture conditions (Fig. 5B). Specifically, the removal of WNT3A combined with Wnt inhibition by DKK1 (−WNT +RSPO +DKK1) had the highest number of unique DEGs (265), while the sole removal of RSPO (−RSPO +WNT3A) had the lowest number of unique DEGs (40). Removal of both WNT3A and RSPO (−WNT −RSPO) was more similar to −WNT +RSPO +DKK1 conditions (Fig. 5B). Moreover, almost 50% of the DEGs were shared between at least two conditions, indicating that WNT3A and RSPO have a shared downstream gene network and that their level of transcription could vary based on the combination of factors present in the culture medium (Fig. 5B).

DEG analyses revealed that the most highly induced gene in the absence of WNT3A (−WNT +RSPO +DKK1) and in the absence of RSPO (−RSPO +WNT), compared to control, is ATF5 (Fig. 5C), a transcription factor known to induce cell cycle arrest of hepatic cells (Gho et al., 2008). Furthermore, shared DEGs between the three conditions compared to control included genes known to play a key role in quiescence, such as *CDKN1A* (p21) or stress response and apoptosis such as *CLU* (Rizzi et al., 2009). These genes are known to be downstream of pathways related to EGFR and KRAS for cell proliferation and BAX for apoptosis, as illustrated in our gene regulatory network analyses (Fig. S4). Interestingly, absence of Wnt and/or RSPO also significantly downregulated *MYC* (Fig. 5D), a factor known to promote proliferation (Cairo et al., 2008). Thus, MYC could be the downstream effector of Wnt signalling, maintaining the proliferative capacity of hepatoblasts. These data also confirmed that absence of WNT3A does not result in differentiation since the expression of markers for hepatocyte or cholangiocyte lineages was not affected (Fig. 1).

Next, we decided to investigate the role of ATF5 in hepatoblasts by overexpression (OE). For this, HBOs were grown in the presence of lipid nanoparticles (LNPs) encapsulating *GFP*-mRNA as a control and *ATF5/GFP*-mRNAs for *ATF5* OE (Fig. 5E). GFP-positive cells were observed in both control HBOs and ATF5 OE 7 days after transfection, confirming the efficacy of LNPs to transfect HBOs. Moreover, significant upregulation of *ATF5* was detected by qPCR in *ATF5* OE HBOs compared to control (Fig. 5E,F). Next, we evaluated key proliferative markers 7 days after transfection. Interestingly, we found that the proliferative marker *MKI67* and the Wnt target gene *LGR5* were significantly downregulated after *ATF5* OE (Fig. 5F), indicating that increased *ATF5* expression can limit hepatoblast proliferation. Furthermore, we evaluated the effect of *ATF5* OE in hepatoblast (ALB, AFP), hepatocyte (ALB, MRP2, G6PC, C3) and biliary (CK19, SOX9, HNF6) markers. We revealed that *ATF5* expression did not impact hepatic markers at the transcript and protein levels (Fig. 5F,G). However, CK19 and SOX9 were significantly downregulated upon *ATF5* OE, although their levels were low and we could not detect CK19 at the protein level (Fig. 5F,G). Taken together, these observations indicate that decreased WNT3A and/or RSPO activity result in the induction of genes involved in cell cycle arrest and stress response. These results also confirm that Wnt signalling is not necessary to maintain the gene expression profile characterising hepatoblast identity or to block differentiation, but rather to protect their self-renewal capacity.

### Single-cell transcriptomic analyses confirm that Wnt activity correlates with hepatoblast proliferation and differentiation during *in vivo* human liver development

**Fig. 6.**
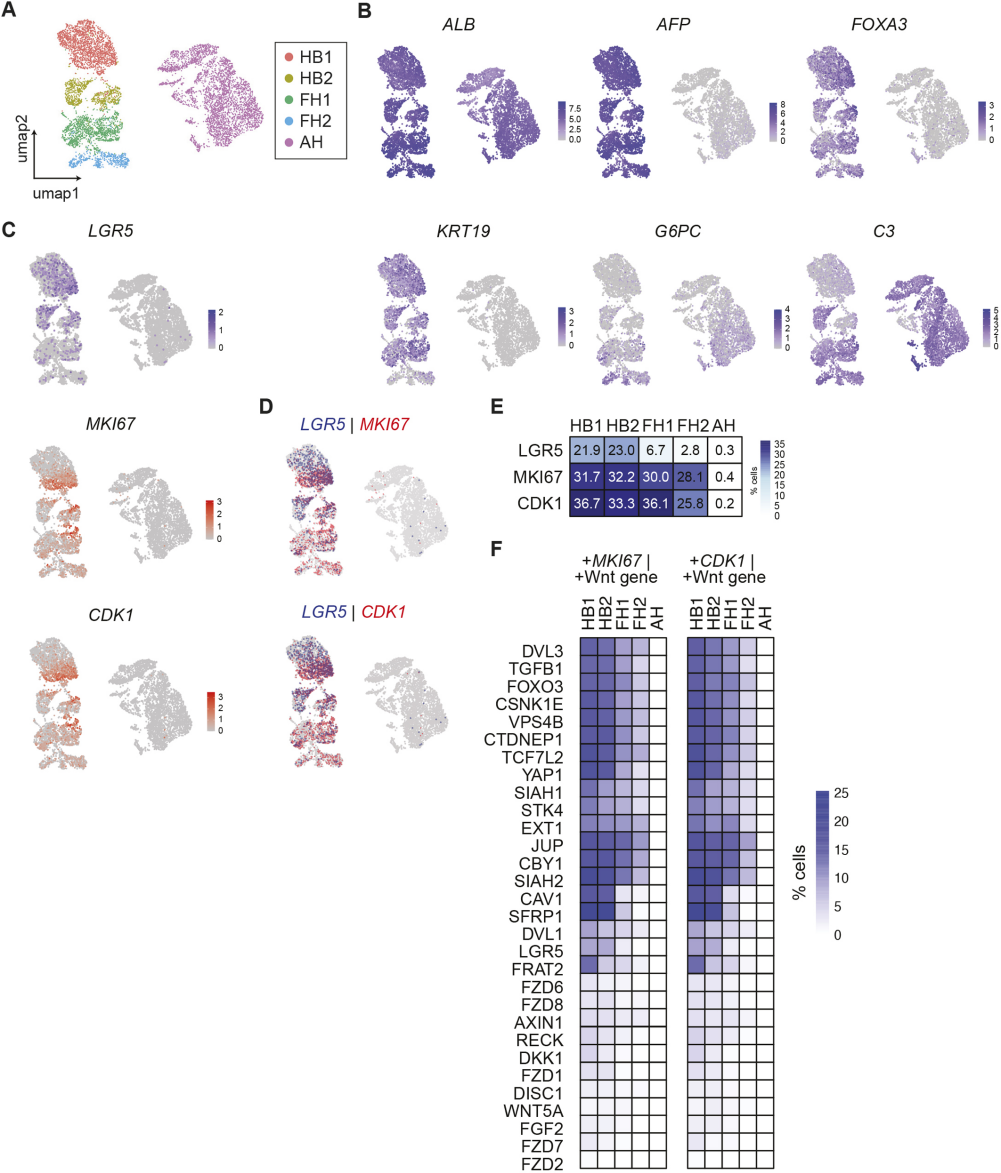
**Active Wnt signalling matches proliferation changes through liver development.** (A) UMAP plot of single-cell RNA-seq data (Wesley et al., 2022), clustering cells by gene signature through liver development. Each dot represents one cell. AH, adult hepatocyte cluster (one adult liver sample); FH1/2, foetal hepatocyte clusters 1 and 2 (FH1 from 7, 8, 9 and 11 pcw samples; FH2 from 12, 14, two 16, and 17 pcw samples); HB1/2, hepatoblast clusters 1 and 2 (HB1 derived from a 5 pcw sample; HB2 from a 6 pcw sample). (B,C) UMAP plots showing distribution of gene expression for hepatoblast markers (*ALB*, *AFP*, *FOXA3*), a biliary marker (*CK19*) and mature hepatic markers (*G6PC*, *C3*) (B); and the Wnt target gene *LGR5*, the proliferation marker *MKI67* and the cell cycle gene *CDK1* (C). (D) UMAP plots showing co-expression of *LGR5*, denoting active Wnt signalling, versus the cell cycle/proliferation genes *MKI67* and *CDK1*. (E) Percentage of cells positive for the denoted genes in each cluster. (F) Percentage of cells co-expressing Wnt-targeted genes and *MKI67* (left) or *CDK1* (right) in each cluster.

To confirm that our results generated *in vitro* could be relevant for natural development, we took advantage of our single-cell atlas of human liver development (Wesley et al., 2022). These analyses revealed the developmental trajectories of hepatocytes and the different steps leading to their functional maturation. In sum, we were able to detect two hepatoblast stages (HB1 and HB2), followed by two successive foetal hepatocyte stages (FH1 and FH2), characterised by the acquisition of key hepatic functions with gradual loss of foetal hepatoblast markers, such as *AFP* and *FOXA3* and the biliary marker *CK19*, and one adult hepatocyte stage (AH) (Fig. 6A,B; Fig. S5A). Using this dataset, we defined the correlation between the Wnt target gene *LGR5* expression and cellular proliferation markers, such as *MKI67* or *CDK1* (Fig. 6C-D and Fig. S5B). We found that *LGR5* is mainly expressed in the hepatoblast clusters *in vivo*, confirming that Wnt signalling is likely to play a key role in self-renewal in the early liver (Fig. 6C). However, *LGR5* activity is not required for later stages of development, especially during functional maturation of foetal hepatocytes (Fig. 6C). Interestingly, the number of cells expressing proliferation markers appears to remain constant during development while they almost disappear in the adult liver (Fig. 6E). However, the highest level of proliferation markers was observed in the hepatoblast stages HB1 and HB2, when Wnt is the most active, based on expression of *LGR5* (Fig. 6D-E). Thus, these observations suggest that Wnt activity correlates with the highest level of proliferation markers in hepatoblasts *in vivo*. This result was further validated by showing that these observations apply not only to the Wnt target gene *LGR5*, but also to 29 known Wnt effector genes, expression of which decreases as development progresses and which are co-expressed with the cell cycle–associated genes *MKI67* and *CDK1* (Fig. 6F; Fig. S6A). These findings support a correlated decline of Wnt signalling and proliferation during hepatoblast *in vivo* development. Of note, we also analysed the expression of identified genes in our scRNA-Seq analyses, and we observed that *ATF5* and *CLU* increase during progression of liver development (Fig. S5C), thereby indicating that induction of these genes is associated with functional maturation of hepatocytes. On the other hand, *CDKN1a* was not induced during human liver development, and thus its expression could be more specific to *in vitro* culture (Fig. S5C). To conclude, our analyses show that Wnt activity correlates with proliferation of hepatoblasts *in vivo*, indicating that this signalling pathway could be essential for self-renewal of hepatoblasts not only *in vitro*, but also during human liver development.

## DISCUSSION

In this study, we utilised a novel cell model system based on primary human HBOs (Wesley et al., 2022) to dissect the role of the Wnt signalling pathway in early human liver development. Wnt activation has been shown to be important for hepatoblast proliferation and maturation in mice (Monga et al., 2003; Micsenyi et al., 2004; Tan et al., 2008). However, how Wnt enables these contradictory roles have not been comprehensively defined, particularly in humans. Here, we have demonstrated that HBOs do not undergo differentiation into hepatocytes or cholangiocytes upon absence of Wnt activation. Instead, removal of Wnt activators decreases the proliferative capacity of HBOs. These results reinforce previous studies showing that overexpression of Wnt inhibitor DKK1 produced smaller than normal livers during chick development (Suksaweang et al., 2004), thereby suggesting that the role of Wnt during early liver organogenesis could be conserved between species. Furthermore, we also found that presence of Wnt does not block differentiation toward either the hepatocyte or cholangiocyte lineages. However, hepatoblast differentiated into the hepatocyte lineage in the presence of Wnt activators do not fully commit to their new lineage, evidenced by their capacity to reactivate the expression of hepatoblast markers such as AFP. These results suggest that Wnt signalling does not act as a conventional factor maintaining (multi)potency, such as LIF or TGFβ/Nodal/Activin signalling, which block the expression of differentiation markers and/or maintain the transcriptional network characterising their corresponding stem cells (Smith et al., 1988, 2008; James et al., 2005; Vallier et al., 2005; Niwa et al., 2009; Osnato et al., 2021). Instead, we found that Wnt signalling is neither sufficient nor necessary to maintain the expression of hepatoblast markers.

Importantly, we have demonstrated that Wnt signalling maintains long-term HBO self-renewal and viability by protecting proliferation. Specifically, we showed that Wnt sustains proliferation through the inhibition of cell cycle inhibitors, including CDKN1a or ATF5. Indeed, we found that long-term absence of Wnt decreases self-renewal and viability of HBOs. Interestingly, we also found a decrease of proliferation associated with differentiation of hepatoblast into hepatocyte *in vivo* and their subsequent functional maturation. Thus, we hypothesise that a decrease in Wnt signalling activity during development could slow down cell cycle progression, allowing hepatoblasts to receive inductive differentiation signals, such as TGFβ for cholangiocytes specification (Carpentier et al., 2011). This would imply that cell cycle progression is not compatible with cell fate commitment, as suggested by our observations *in vitro*. Additionally, Wnt signalling may influence the temporal differentiation of hepatoblast during their proliferative phase by allowing the expression of specific receptors that prime them for subsequent hepatocyte or cholangiocyte differentiation. Interestingly, Wnt signalling is known to drive adult liver regeneration (Monga et al., 2001; Nelsen et al., 2001), suggesting shared mechanisms in liver development and regeneration. Further studies will uncover the molecular interplays between Wnt signalling, cell cycle regulators and factors driving differentiation.

Wnt signalling is known to drive liver zonation *in vivo*, where hepatocytes show opposing metabolic processes depending on their location in the liver (Jho et al., 2002; Sekine et al., 2006; Tan et al., 2006; Gougelet et al., 2014). Hepatocytes surrounding the pericentral vein show an active Wnt pathway, while as they move away, their Wnt activation decreases (Benhamouche et al., 2006). Therefore, the role of Wnt could vary in function of the developmental stage and the location of the cells in the liver. This hypothesis could explain in part why previous studies have generated contradictory observations regarding the function of Ctnnb1 in mouse liver development (Tan et al., 2008; Decaens et al., 2007).

We can also distinguish between hepatocyte and cholangiocyte differentiations. Based on our result, Wnt is unlikely to have the same impact on both cell fates. First, absence of Wnt has no effect on hepatocyte differentiation while it seems to result in the induction of biliary markers in hepatoblasts. Interestingly, Wnt is known to interact with TGFβ in different contexts (Ying et al., 2003; Lei et al., 2004; Luo, 2017). Thus, we would like to propose that decrease in Wnt signalling could prime hepatoblasts toward the biliary fate resulting in the formation of the ductular plate *in vivo*. Then, Wnt could synergise with TGFβ signalling to ensure cholangiocytes specification. This model could explain why Wnt seems to suppress the hepatic genetic programme in hepatoblasts and instead drives them towards the biliary fate for ductal plate remodelling *in vivo* (Decaens et al., 2007).

Taken together, our results propose a new model concerning the function of Wnt in hepatoblasts which would principally protect cell cycle progression, and in turn will maintain differentiation capacity. In parallel, Wnt signalling could also cooperate with inductive signals of differentiation to promote the commitment of hepatoblasts toward specific lineages. We suspect that this model could apply to a broad diversity of stem cells and adult cells which rely on Wnt signalling.

## MATERIALS AND METHODS

### Media formulation

See Table S1 for details of media composition and formulation.

### Generation and maintenance of HBO lines

HBOs were established and cultured following conditions developed by Wesley et al. (2022). Briefly, HBOs were generated from embryonic human foetal livers aged between 5 and 10 pcw. Primary human foetal tissue was obtained from individuals undergoing elective terminations (ethical approval obtained from Cambridge Central Research Ethics Committee REC-653 96/085). The primary tissue was dissected and immediately washed in Hank's Balanced Salt Solution (HBSS; Fisher Scientific, 10617394) and transferred to a pre-warmed solution containing 1.07 Wünsch units/ml Liberase DH (Sigma-Aldrich, 5401054001) with 70 U/ml hyaluronidase (Sigma-Aldrich, H1115000) diluted in HBSS. The tissue was then incubated at 37°C on a microplate shaker at 750 rpm for 10-15 min. The dissociated tissue was washed four times with HBSS at 400 ***g*** for 5 min, the supernatant was discarded and the single cells were resuspended in 45% complete HBO media and 55% growth-factor reduced Phenol Red-free Matrigel (Corning, 356231) and seeded in 20 μl domes on 48-well plates (Corning, 3548). Plates were incubated at 37°C for 10-15 min. Following incubation, 200 μl of fresh HBO complete media was added. Medium was changed every second day. To reduce the presence of red blood cells and select for hepatoblasts, following enzymatic dissociation the tissue can be sorted on a magnetic-activated cell sorting (MACS) machine based on EPCAM expression. HBOs were split every 10-14 days based on size and density of the organoids at a split ratio of 1:2 to 1:3 for up to 25 passages. For splitting, organoids were collected to 1.5 ml LoBind tubes (SLS, 30108051) and incubated on ice to dissolve the Matrigel for up to 30 min based on the number of wells collected. The organoid suspension was pipetted up and down 10-20 times to reach a clump size of approximately six to eight cells and centrifuged at 400 ***g*** for 5 min at 4°C to pellet the organoids. The supernatant was removed and organoids were resuspended and seeded as explained above. HBOs were frozen when they were confluent and were relatively big in size using Cell Banker (Fisher Scientific, 11891). HBOs tested negative for *Mycoplasma* contamination. To increase Wnt signalling, complete HBO medium was supplemented with 3 μM CHIR99021 (Tocris, 4423/10). To reduce Wnt signalling, Wnt3a and R-spondin conditioned media were removed alone or in combination and replaced by HBO basal media (Table S1); alternatively, 2.5 µM DKK1 Wnt inhibitor (Abcam, ab155623) was added to the medium. Treatments were performed on at least three different HBO lines (from three different donors) and grown in complete HBO medium. Treatments lasted for 7 days. Cells were kept in culture and split in the reduced Wnt conditions for up to two passages. Conditions tested are summarised in Table 1.

**Table 1.** Reduced Wnt conditions investigated

Condition	Function
	
HBO maintenance+3 µM CHIR99021	Wnt activation
HBO maintenance without Wnt3a CM	Wnt removal, R-spondin present
HBO maintenance without Wnt3a CM+2.5 µM DKK1	Wnt removal, Wnt inhibition, R-spondin present
HBO maintenance without R-spondin CM	R-spondin removal, Wnt present
HBO maintenance without Wnt3a CM and R-spondin CM	Combined Wnt and R-spondin removal

CM, conditioned medium.

### HBO differentiation into hepatocytes and cholangiocytes

HBOs were differentiated to either hepatocytes or cholangiocytes based on established protocols described by Wesley et al. (2022). For hepatocyte differentiation, complete HBO media was removed and replaced with HepatoZYME-SFM Complete medium supplemented with 20 ng/ml oncostatin M (Peprotech, 300-10T). For cholangiocyte differentiation, HBO media was removed and replaced with complete HBO media to which A83-01 was subtracted from and supplemented with 2 ng/ml TGFβ (R&D Systems, 240-B-500/CF). Treatment lasted for 7 days, and differentiation media were changed every second day.

### Live/dead assay on HBOs

Live/dead assay was performed with a commercially available kit (LIVE/DEAD Cell imaging kit 488/570, Fisher Scientific, R37601) following the manufacturer's instructions. The treated organoids were then visualised using an EVOS inverted microscope.

### Immunofluorescence microscopy

All antibodies used are listed in Table S2. Immunocytochemistry was performed on both 2D cells on plastic and 3D organoid cultures. For 2D cultures, cells were fixed in 4% paraformaldehyde (Thermo Fisher Scientific, 043368.9M) in PBS (Gibco, 14190169) for 20 min at 4°C. Following incubation, cells were washed three times with PBS and stored in PBS at 4°C until further staining.

For intracellular proteins, cells were permeabilised using 10% donkey serum (Bio-Rad, C06SB) in PBS+0.1% Triton X-100 (Sigma-Aldrich, T8787) for 30 min at room temperature (RT). Primary antibodies were diluted according to manufacturer's instructions in PBS + 1% donkey serum+0.1% Triton X-100 and samples were incubated at 4°C overnight. After three washes with PBS, samples were incubated with secondary antibody in PBS + 1% donkey serum+0.1% Triton X-100 (diluted 1:1000) for up to 2 h at RT. Cells were washed once and nuclei were stained with 1:10,000 Hoechst 33258 (Sigma-Aldrich, b2883-25MG) in PBS. Cells were washed twice and stored in PBS until imaging.

For 3D organoid staining, cells were washed with PBS once and incubated with 4% paraformaldehyde in PBS for 20 min at RT, followed by three washes with PBS then storage in PBS at 4°C. For intracellular epitopes, organoids were permeabilised using a solution of 10% donkey serum in PBS + 0.3% Triton X-100 for at least 3 h. Cells were incubated with primary antibody in PBS + 1% donkey serum+0.1% Triton X-100 (diluted according to the manufacturer's instructions) at 4°C overnight. Cells were washed with PBS three times at RT for 1 h per wash. Then, cells were incubated with secondary antibody diluted 1:1000 in PBS + 1% donkey serum+0.1% Triton X-100 at 4°C overnight. Cells were washed with PBS three times at RT for 1 h per wash. Cells were stained with 10 mg/ml Hoechst dye (1:10,000 in PBS) for 30 min and washed twice. Cells were stored in PBS at 4°C for up to 1 month.

Fluorescent images were captured using a Zeiss Axiovert 200M or a Zeiss LSM880-1 Airyscan confocal microscope. Optical sections with a thickness of 2.5 μm along the *z*-axis were collected. The images were processed in Fiji.

Limited antibody penetration likely explains the apparent absence of some markers, such as ALB, in the centre of HBOs. For this reason, we systematically combined immunostaining with qPCR to support our conclusions.

### Immunohistochemistry on paraffin-embedded tissue

Foetal liver tissues were fixed in 10% neutral buffer formalin for 24 h at 4°C. Following two washes with 70% ethanol, the fixed tissue was stored in 70% ethanol at 4°C, prior to embedding it in paraffin. Paraffin-embedding and sectioning were performed by the CSCI Histology Facility. Prior to staining, the antigen retrieval buffer (1 mM EDTA, pH 8; Sigma-Aldrich, E9884-100G) was pre-heated in a steamer for 30 min. For immunohistochemistry, slides were de-paraffinised by two 10-min washes in HistoClear II (SLS, NAT1334). To re-hydrate the tissue, slides were placed in 100% ethanol and washed three times, 5 min per wash. To remove any remaining ethanol, slides were placed under running tap water for 5 min. The slides were incubated in the pre-heated antigen retrieval buffer in the steamer for 20 min and were cooled down at RT for 20 min. To remove any remaining buffer, slides were placed under running tap water for 5 min. Then, slides were washed once with PBS + 0.1% Triton X-100 (Sigma-Aldrich) for 5 min at RT. The slides were dried without interfering with the tissue and an ImmEdge Hydrophobic PAP pen (Biotechne, 310018) was used to demarcate the perimeter of the tissue. The slides were then placed in a wet chamber and tissue was blocked in 10% donkey serum (Bio-Rad) in PBS+0.1% Triton X-100 for 1 h at RT. Primary antibody was diluted in PBS+1% donkey serum+0.1% Triton X-100 and placed on the tissue overnight at 4°C in the wet chamber. After three washes with PBS+0.1% Triton X-100, 5 min per wash, secondary antibody diluted in PBS+1% donkey serum+0.1% Triton X-100, was placed on the tissue and incubated for 1 h at RT in the dark. The tissue was then washed twice at RT with PBS+0.1% Triton X-100, 5 min per wash, and incubated for 5 min at RT in DAPI (1:10,000 mg/ml; Thermo Fisher Scientific, 62248). Following incubation, the tissue was washed twice with PBS at RT, 5 min per wash. Samples were mounted with Fluoromount-GTM Mounting Medium (Invitrogen, 00- 4958-02). Antibody information is shown in Table S2.

### Western blotting

Cell pellets were incubated with 0.5 ml RIPA Buffer, supplemented with 1× protease inhibitor (Roche, 5892791001) and 1× phosphatase inhibitor (Merck, 4906837001) and centrifuged at 14,000 rpm (400 ***g***) for 5 min at 4°C. The supernatants were collected and diluted 1:5 in PBS. To quantify the total protein content, samples for a standard curve were prepared with a BCA protein assay kit (Thermo Fisher Scientific, 23227) according to the manufacturer's instructions and using a Perkin Elmer EnVision plate reader. Equal amounts of protein per sample were then diluted in 4× NuPAGE LDS Sample Buffer (Thermo Fisher Scientific, NP0007) with 4% β-mercaptoethanol (Sigma-Aldrich), spun down and heated at 95°C for 5 min. Samples were placed in the Xcell SureLock MiniCell (Thermo Fisher Scientific) and were electrophoresed with 5 μl of the Precision Plus ladder (Bio-Rad, 161-0374), using pre-cast NuPAGE 4-12% gels (Thermo Fisher Scientific, NP0321BOX) and 1× MOPS Buffer (Thermo Fisher Scientific, NP0001). The proteins were run on the chamber using a 200 V and 130 mA current for 45-60 min. Gels were transferred to PVDF membranes (Bio-Rad, 162-0177) in Mini Trans-Blot Cell (Bio-Rad) with 1× NuPAGE Transfer Buffer and methanol. To transfer the membrane, a 200 V, 180 mA current was applied for at least 1 h. The membranes were blocked with PBS with 0.1% Triton (PBS-T) with 4% milk (commercial, blocking solution) for 1 h at RT, followed by overnight incubation at 4°C with the blocking solution containing the primary antibody and 1 h incubation at RT with the secondary antibody diluted in the blocking solution (Table S4). After both incubations, the membrane was washed thrice with PBS-T. All incubations were carried out in a rotator. Finally, signals were detected with the ECL detection kit (Thermo Fisher Scientific, PN80196) and the CL-Xposure film 18×24 cm (Thermo Fisher Scientific, 34089).

### RNA extraction

Total RNA was extracted from cells using the GenElute total RNA purification kit (Sigma-Aldrich, RTN350) according to the manufacturer's instructions including the on-column treatment with DNase I (Sigma-Aldrich, DNASE70) digestion before elution to reduce DNA contamination.

### Reverse transcription

Five-hundred nanograms of total RNA was added to 250 µg/µl of random primers (Promega, C1181) and 0.5 mM dNTPs (Promega, U1511). This mixture was heated to 65°C for 5 min and immediately transferred to ice. Reverse transcript master mix was prepared with 5×1st Strand buffer (Invitrogen), 0.01 M DTT (Sigma-Aldrich, 43815), 25 U Superscript II Reverse Transcriptase (Thermo Fisher Scientific, 18064071) and 20 U RNase OUT (Invitrogen, 10777019), and 6.7 µl of the reverse transcript master mix were added into each sample. Samples were incubated at 25°C for 10 min for the primer annealing step, followed by extension at 42°C for 50 min and enzyme inactivation at 70°C for 15 min. The obtained cDNAs were diluted 30-fold using nuclease-free water before proceeding to the next step.

### Quantitative PCR

To perform quantitative PCR, KAPA SYBR FAST qPCR Mastermix (2×) kit (Kapa Biosystems, KK4601) was used with a 10 μM mix of reverse and forward primers. Technical duplicates were prepared for each biological triplicate in a 384-well qPCR plate (Thermo Fisher Scientific, AB1384), loaded on a QuantStudio 5 Real-Time PCR system (Applied Bioscience). All data were analysed using the 2−ΔCt method and normalised to the expression level of the housekeeping genes porphobilinogen deaminase (*PBGD*; also known as *HMBS*) and ribosomal protein lateral stalk subunit 0 (*RPLP0*). Primer sequences are listed in Table S3.

### Transfection of HBOs with LNPs

LNPs were acquired from VectorBuilder. Transfection of LNPs to HBOs was carried out during splitting; HBOs were dissociated by mechanical dissociation as usual and were seeded in 1:2 HBOc medium:Matrigel domes containing 0.4 μg of LNPs. HBOc medium containing 0.4 μg of LNPs was added on top after Matrigel was solidified. Medium was refreshed every other day without LNPs.

### RNA-seq library preparation

RNA-seq libraries were prepared at the Gene Service Facility (Cambridge Stem Cell institute). Initial quality control of the RNA was carried out with the Qubit RNA HS Assay Kit (Q32855), and either Agilent RNA TapeStation reagents (5067-5576; 5067-5577; 5067-5578) or with the Agilent Bioanalyzer kit (RNA 6000 Nano Kit; 5067-1511). After confirming that all RIN values were 9 or higher, libraries were prepared starting with 200-300 ng input RNA and using NEBNext^®^ Ultra™ II Directional RNA Library Prep Kit (Illumina, E7760) and the NEBNext^®^ Multiplex Oligos 96 Unique Dual Index Primer Pairs (Illumina, E6440) in conjunction with Poly(A) mRNA Magnetic Isolation Module (Illumina, E7490), following the manufacturer's instructions. Quality control of the libraries was carried out with Qubit dsDNA HS Assay Kit (Q32854) and Agilent DNA 5000 TapeStation reagents (5067-5588; 5067-5589). Samples were pooled in equimolar quantities. Sequencing was performed at the CRUK (Cancer Research UK) Cambridge Institute Genomics Core Facility on a Novaseq6000 sequencer as paired end 100 bp (PE100) reads.

### RNA-seq analysis

Raw fastq files were quality checked with FastQC (v.0.11.8) and results summarised with MultiQC (v.1.9). Checks included nucleotide composition, GC-content distribution, and adapter contamination. Because of a large variation in sequencing depths, per sample, all samples were subsampled to 30 million reads (Mohorianu et al., 2017), the resulting fastq files were aligned to the human GRCh38.p13 reference genome using STAR (v.2.7.6a) with default parameters (Dobin et al., 2013). The count table was generated using featureCounts (v.2.0) (Liao et al., 2014) with ENSEMBL (v.101) annotations.

Further quality control checks were performed, i.e. density of gene expression and pairwise sample MA-plots. Subsequently, a noise filter was applied to reduce the influence of low-abundance genes (Moutsopoulos et al., 2023). Specifically, genes with <20 counts across all samples were discarded from downstream analysis; 11,989 genes total were retained. Subsequently, the gene expression levels were normalised using quantile normalisation (Bolstad et al., 2003). The quality of the normalised data was assessed using hierarchical clusterings, PCAs and Jaccard similarity heatmaps, all created on the [50, 100, 250, 500, 1000, 2000] most abundant genes, as well as a PCA created on a curated list of cell-cycle genes (Tirosh et al., 2016).

Differential gene expression analysis was performed using edgeR (v.3.26.8) (Robinson et al., 2010) on the noise-corrected, normalised expression levels. Comparisons comprised control versus each of the treatments: −RSPO−WNT, −RSPO+WNT and −WNT +DKK1, as well as pairwise comparisons between the treatments. Genes with Benjamini–Hochberg-corrected *P*<0.05 and |log2(FC)|>0.5 between groups were considered DEGs. DEG comparisons were contrasted using cross plots of log2 fold-changes on the set of DEGs in at least one comparison. DEGs were further assessed in a gene set enrichment analysis with g:Profiler (Reimand et al., 2016), using data sources GO, KEGG, Reactome, TF, with all genes passing the noise threshold used as background.

## Supplementary Material

10.1242/develop.205026_sup1 Supplementary information
