# Supplementary material for: Wnt signalling maintains self-renewal of human hepatoblasts without blocking their differentiation
Source: Development. 2025 Nov 20;152(22):dev205026. doi: 10.1242/dev.205026 (PMC12669971; doi:10.1242/dev.205026)
Supplement: Supplementary information [file develop-152-205026-s1.pdf]

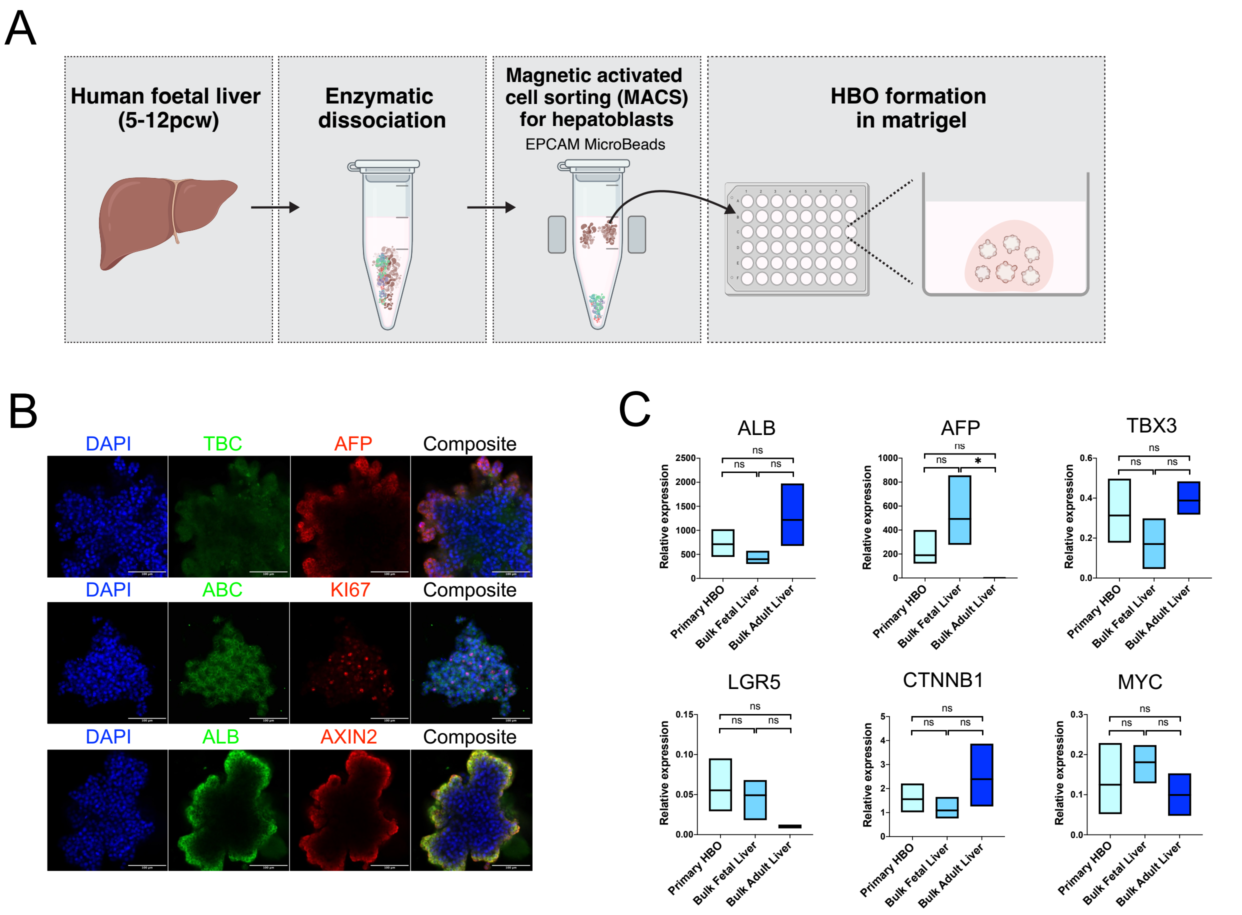


**Fig. S1. HBOs display an active Wnt signalling pathway.** (A) Schematic representation of HBO derivation. (B) Immunofluorescence staining of hepatoblast markers (ALB, AFP), proliferation marker (KI67) and active WNT signalling markers (TBC, ABC and AXIN2). Scale bars: 1000 μm. (C) QPCR showing the expression of denoted genes, including Wnt signalling targets TBX3, LGR5, CTNNB1 and MYC, and the hepatic markers ALB and AFP from primary HBOs (n=5), bulk foetal (n=3) and adult liver (n=3). Data are shown as means ± s.d., *$P < 0.05$ (one-way ANOVA).

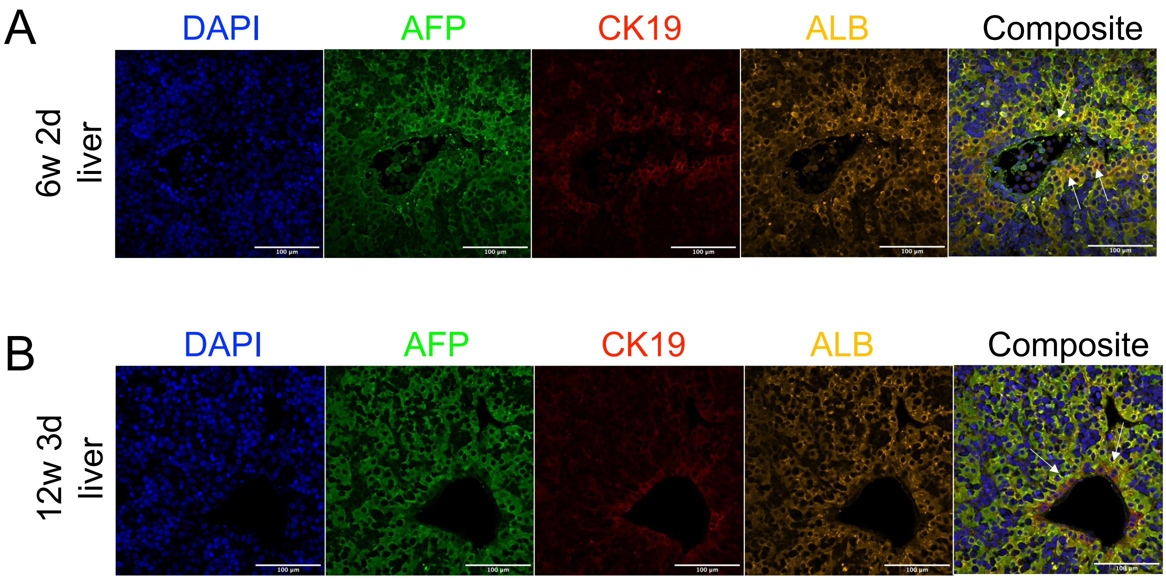


**Fig. S2. Ductal plate progenitors express markers of hepatic and biliary lineages.** Immuno-histochemistry staining of paraffinized liver tissue from an early embryo aged 6 weeks and 2 days (6w 2d, A) and a late embryo aged 12 weeks and 3 days (12w 3d, B) with hepatic markers (ALB, AFP) and biliary marker (CK19). White arrows indicate the cells that are co-expressing ALB and CK19 during early liver development surrounding vessels that denote the ductal plate progenitors (A). As development progresses, ALB expression decreases and CK19+ cells localise only around vessels (B). Scale bars: 100 µm.

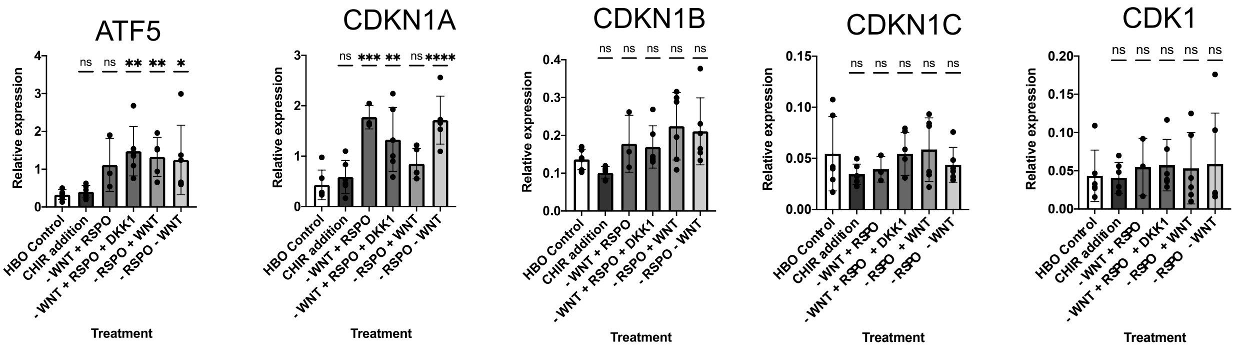


**Fig. S3. Absence of Wnt signalling has little effect on cell cycle regulators in HBOs.** QPCR analyses for the expression of genes inhibiting (ATF5, CDKN1A, CDKN1B, CDKN1C) and promoting (CDK1) cell cycle. Data are shown as means ± s.d. in six to three independent biological replicates (n=6-3), where *P < 0.05, **P<0.01, ***P<0.001, ****P<0.0001 (one-way ANOVA).

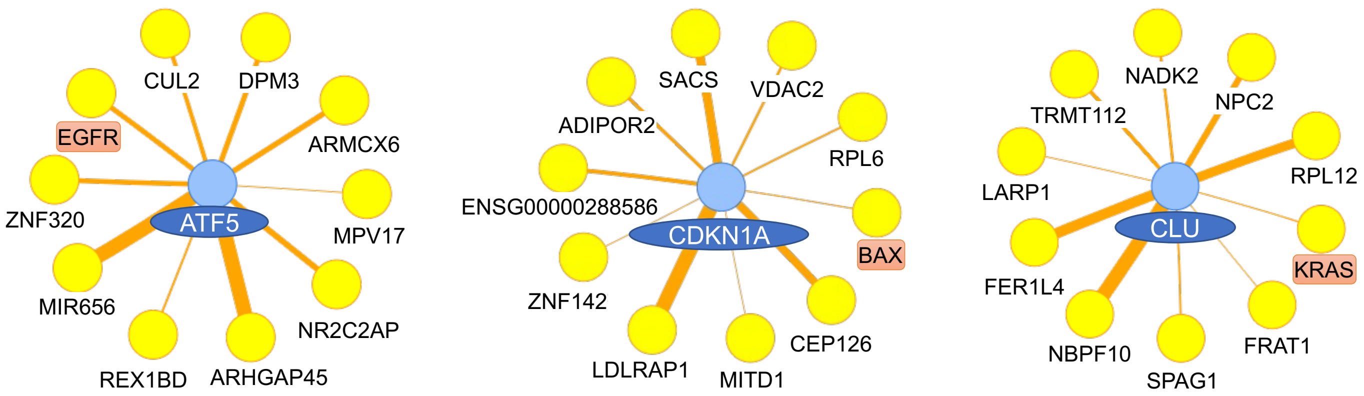


**Fig. S4. Common DEG regulated by other signalling pathways.** Gene regulatory networks inferred from bulk RNA-seq data for upstream regulators of common DEG hits. Cell cycle and apoptosis regulators affected by common growth signalling pathways (EGFR, KRAS) and apoptosis proteins (BAX).

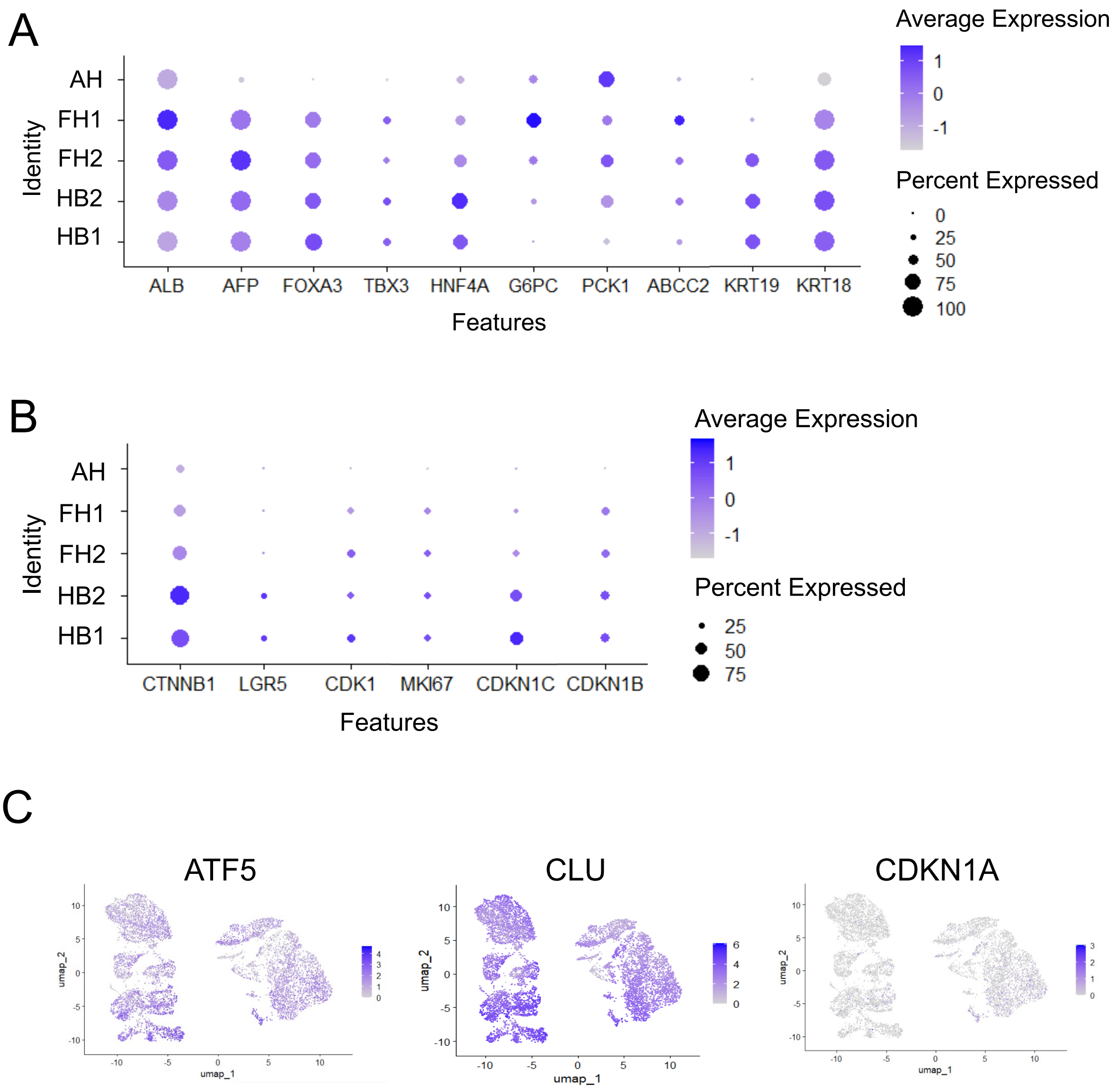


**Fig. S5. Progression of liver development by marker expression.** (A, B) Changes in gene expression level and strength through liver development in each cluster for hepatoblast, hepatic and biliary genes (A) and Wnt target genes (B). (C) UMAP plots showing distribution of RNA-seq DEG hits.

Development • Supplementary information

**A**

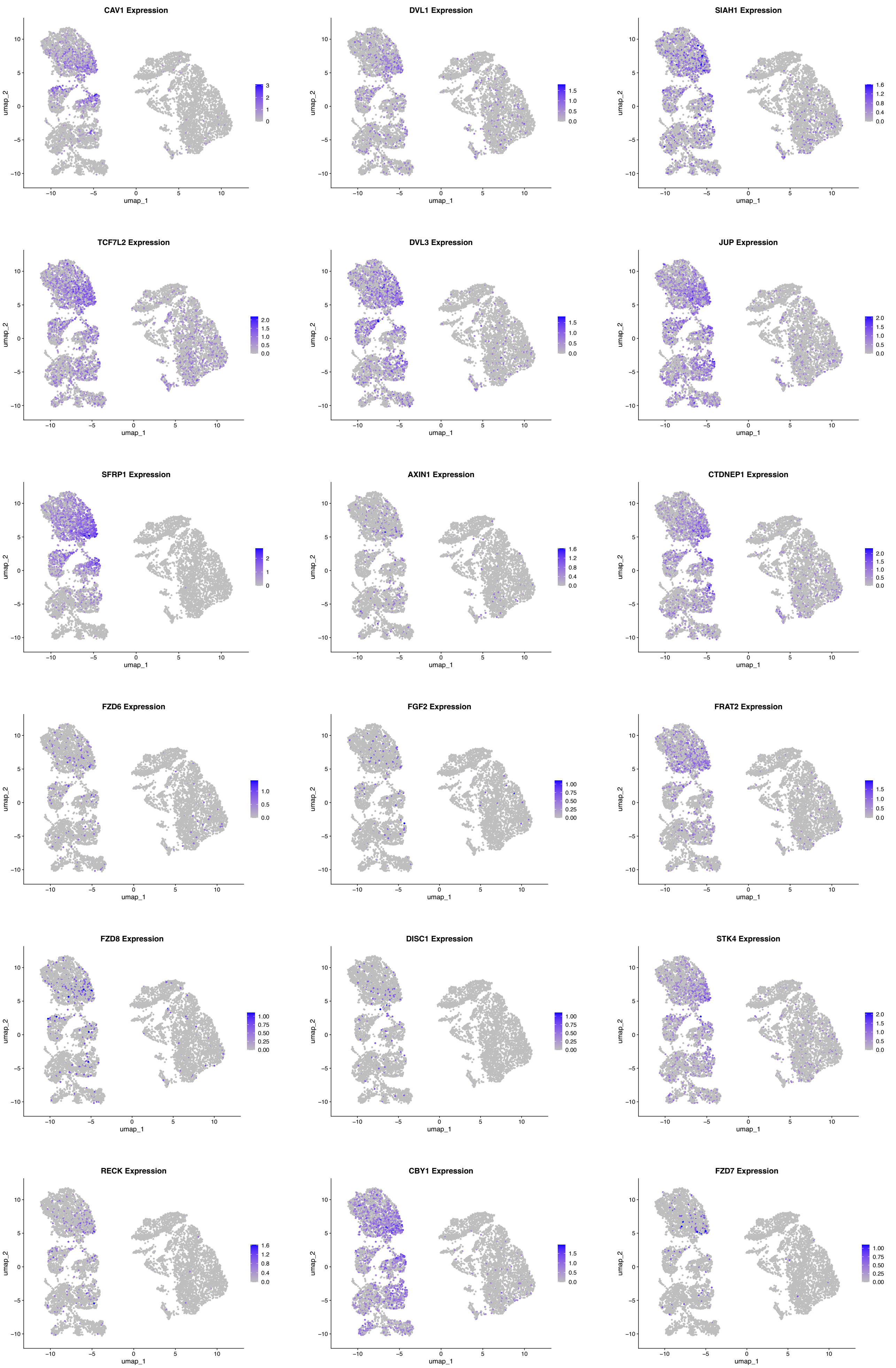


Development • Supplementary information

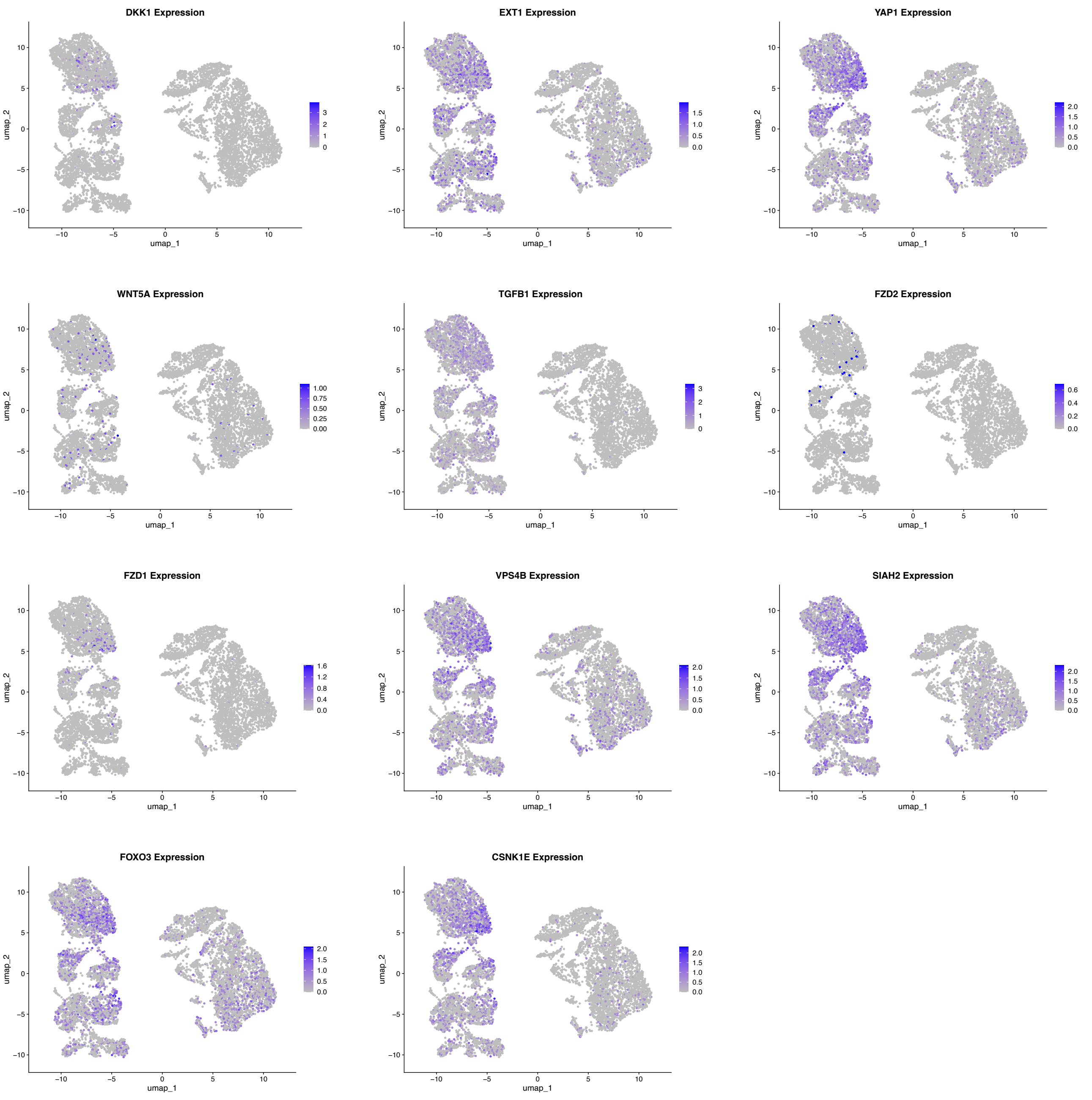


**Fig. S6.** UMAP plots showing gene expression distribution for Wnt  effectors during human liver development from hepatoblast to adult hepatocyte stage.

Development • Supplementary information

## Table S1. Media formulation

| HepatoZYME-SFM Complete Medium | | | | |
|---|---|---|---|---|
| ***Company*** | ***Cat No*** | ***Name*** | ***Amount*** | ***Concentration*** |
| ThermoFisher | 11705-021 | HepatoZYME-SFM | 500 mL | - |
| ThermoFisher | 17504044 | Non-essential amino acids | 10 mL | 2% |
| ThermoFisher | 11140050 | Concentrated Lipids | 10 mL | 2% |
| ThermoFisher | 25030024 | L-Glutamine | 5 mL | 1% |
| Roche | 652202 | Transferrin | 500 µL | 30 µg/mL |
| Roche | 1376497 | Insulin | 700 µL | 14 µg/mL |
| ThermoFisher | 15140122 | Pen/Strep (optional) | 5 mL | 1% |

| Hepatoblast Organoid Maintenance Medium | | | | |
|---|---|---|---|---|
| ***Company*** | ***Cat No*** | ***Name*** | ***Amount*** | ***Concentration*** |
| **Basal Medium (200 mL)** | | | | |
| ThermoFisher | 11320033 | DMEM-F12 | 194 mL | - |
| Sigma | H0887 | HEPES solution | 2 mL | 1% |
| ThermoFisher | 35050061 | GlutaMAX | 2 mL | 1% |
| Core Facility | - | Pen/Strep (optional) | 2 mL | 1% |
| **Complete Medium (10 mL)** | | | | |
| - | - | HBO Basal Medium | 3.6 mL | 36% |
| ThermoFisher | 17504044 | B-27 Supplement (50X) | 200 µL | 1X |
| Sigma | N0636 | Nicotinamide | 200 µL | 20 mM |
| Sigma | A9165 | N-acetylcysteine | 40 µL | 0.4% |
| Core Facility | - | Wnt3a Conditioned Medium | 5 mL | 50% |
| Core Facility | - | R-spondin Conditioned Medium | 1mL | 10% |
| R&D | 236-EG | EGF | 5 µL | 50 ng/mL |
| Tocris | 2939 | A83-01 | 10 µL | 5 µM |
| Selleckchem | S1049 | Y27632 | 10 µL | 10 µM |

| Nuclear Extraction Buffer | | | | |
|---|---|---|---|---|
| ***Company*** | ***Cat No*** | ***Name*** | ***Amount*** | ***Concentration*** |
| Merck | C189847 | Milli-Q water | 7.5 mL | - |
| Sigma | H0887 | 1M HEPES pH 7.0 – 7.6 | 200 µL | 20 mM |
| Sigma | P9541 | 1M KCl | 100 µL | 10 mM |
| Sigma | T8787 | Triton X-100 | 10 µL | 0.1% |
| Sigma | G5516 | Glycerol | 2 mL | 20% |
| | | 1 M Spermidine | 5 µL | 0.5 mM |
| Roche | 4693132001 | 50x EDTA-free protease inhibitor | 200 µL | 1x |

| RIPA Buffer | | | | |
|---|---|---|---|---|
| ***Company*** | ***Cat No*** | ***Name*** | ***Amount*** | ***Concentration*** |
| Merck | C189847 | Milli-Q water | 45 mL | - |
| ThermoFisher | AM9760G | 5 M NaCl | 1.5 mL | 150 mM |
| Sigma | T8787 | Triton X-100 | 500 µL | 1% |
| Sigma | D6750-100G | Sodium deoxycholate | 0.25 g | 0.5% |
| ThermoFisher | AM9822 | SDS 10% | 500 µL | 0.1% |
| Merck | 648314 | 1 M Tris pH 8.0 | 2.5 mL | 50 mM |

Development • Supplementary information

| **1X Transfer Buffer** | | | | |
|---|---|---|---|---|
| ***Company*** | ***Cat No*** | ***Name*** | ***Amount*** | ***Concentration*** |
| - | - | Distilled water | 850 mL | - |
| ThermoFisher | NP0006-1 | 20X Transfer Buffer | 50 mL | 1X |
| FisherScientific | 10284580 | Methanol | 100 mL | 10% |

| **PBS-T** | | | | |
|---|---|---|---|---|
| ***Company*** | ***Cat No*** | ***Name*** | ***Amount*** | ***Concentration*** |
| - | - | Distilled water | 1 L | - |
| Sigma | P4417 | PBS tablets | 5 tablets | - |
| Sigma | P9416 | Tween 20 | 1 mL | 0.1% |

Development • Supplementary information

**Table S2.** List of primary and secondary antibodies.

| Target | Dilution | Manufacturer | Cat Number | Antibody |
|---|---|---|---|---|
| ALB | 1:100 | Bethyl | A80-229A | Primary Antibody |
| AFP | 1:100 | Dako | A0008 | |
| CK19 | 1:100 | Abcam | ab7754 | |
| TBX3 | 1:100 | Santa Cruz | sc-17871 | |
| HNF4a | 1:100 | Abcam | ab92378 | |
| SOX9 | 1:100 | R&D Systems | AF3075 | |
| SPINK1 | 1:200 | Abcam | ab203579 | |
| Ki67 | 1:100 | Abcam | ab15580 | |
| CK7 | 1:100 | Abcam | ab68459 | |
| CK18 | 1:100 | Abcam | ab668 | |
| ASGR1 | 1:50 | ThermoFisher | MA1-40244 | |
| A1AT | 1:100 | Abcam | ab19170 | |
| HPX | 1:100 | Abcam | ab124935 | |
| ABC* | 1:200 | Millipore | 05-665 | |
| TBC* | 1:100 | Abcam | ab32572 | |
| AXIN2 | 1:200 | Origene | TA319745 | |
| FOXA3 | 1:50 | Santa Cruz | sc-166703 | |
| HNF6 | 1:50 | Santa Cruz | sc-13050 | |
| MRP2 | 1:100 | Abcam | ab3373 | |
| Anti-rabbit 568 | 1:1000 | ThermoFisher | A10042 | Secondary antibody |
| Anti-goat 488 | 1:1000 | ThermoFisher | A11055 | |
| Anti-goat 568 | 1:1000 | ThermoFisher | A11057 | |
| Anti-mouse 488 | 1:1000<br>1:200 (tissue IF) | ThermoFisher | A21202 | |
| Anti-mouse 647 | 1:1000 | ThermoFisher | A31571 | |
| Anti-rabbit 555 | 1:200 (tissue IF) | ThermoFisher | A31572 | |
| Anti-goat 488 | 1:200 (tissue IF) | ThermoFisher | A21121 | |

*ABC: active beta-catenin, TBC: total beta-catenin

**Table S3. List of primers.**

| Gene | Forward Primer Sequence | Reverse Primer Sequence |
|---|---|---|
| PBGD | GGAGCCATGTCTGGTAACGG | CCACGCGAATCACTCTCATCT |
| RPLP0 | GGCGTCCTCGTGGAAGTAC | GCCTTGCGCATCATGGTGTT |
| TBX3 | TGGAGCCCGAAGAAGAGGTG | TTCGCCTTCCCGACTTGGTA |
| HHEX | GCCCTTTTACATCGAGGACA | AGGGCGAACATTGAGAGCTA |
| PROX1 | TTATCATATTCATCACCCGCAAGAT | TCAGGCATCACTGGACGGTT |
| HNF4a | CATGGCCAAGATTGACAACCT | TTCCCATATGTTCCTGCATCAG |
| HNF1b | GCACCCCTATGAAGACCCAG | GGACTGTCTGGTTGAATTGTCG |
| FOXA3 | ATTCGCCACTCGCTGTCTTT | TTCCCTGAGCTGGGGTGTAG |
| FOXA2 | GGGAGCGGTGAAGATGGA | TCATGTTGCTCACGGAGGAGTA |
| HNF6 | GTGTTGCCTCTATCCTTCCCAT | CGCTCCGCTTAGCAGCAT |
| OC-2 | ATTCCCCCTATGTGGGTCTC | CCACATGGGAACACTCCTCT |
| CYP3A4 | TGTGCCTGAGAACACCAGAG | GTGGTGGAAATAGTCCCGTG |
| CYP3A7 | GAAACACAGATCCCCCTGAA | TCAGGCTCCACTTACGGTCT |
| CK19 | GGATGTGCTGGATGTGAATG | AGCAAGAGCAGCAGAATCAG |
| ALB | CCTTTGGCACAATGAAGTGGGTAACC | CAGCAGTCAGCCATTTCACCATAG |
| AFP | AGAACCTGTCACAAGCTGTG | GACAGCAAGCTGAGGATGTC |
| A1AT | CCACCGCCATCTTCTTCCTGCCTGA | GAGCTTCAGGGGTGCCTCCTCTG |
| SOX9 | CTCTGGAGACTTCTGAACGAGAG | CCTTGAAGATGGCGTTGGGG |
| CK18 | GAGGGCTCAGATCTTCGCAA | CAATCTGCAGAACGATGCGG |
| LGR5 | CTCCCAGGTCTGGTGTGTTG | GAGGTCTAGGTAGGAGGTGAAG |
| MKI67 | GAAAGAGTGGCAACCTGCCTTC | GCACCAAGTTTTACTACATCTGCC |
| HPX | AGCAGTGGATGCTGCCTTTTCC | TTCTCCAGCCGCTTCGGATAAC |
| C3 | GTGGAAATCCGAGCCGTTCTCT | GATGGTTACGGTCTGCTGGTGA |
| MRP2 | GCCAACTTGTGGCTGTGATAGG | ATCCAGGACTGCTGTGGGACAT |
| G6PC | GTGTCCGTGATCGCAGACC | GACGAGGTTGAGCCAGTCTC |
| CDK1 | GGAAACCAGGAAGCCTAGCATC | GGATGATTCAGTGCCATTTTGCC |
| CDKN1A | AGGTGGACCTGGAGACTCTCAG | TCCTCTTGGAGAAGATCAGCCG |
| ATF5 | GCTCGTAGACTATGGGAAACTCC | CATCCAGTCAGAGAAGCCATCAC |
| CDKN2A | CTCGTGCTGATGCTACTGAGGA | GGTCGGCGCAGTTGGGCTCC |
| CDKN1B | ATAAGGAAGCGACCTGCAACCG | TTCTTGGGCGTCTGCTCCACAG |
| CDKN1C | AGATCAGCGCCTGAGAAGTCGT | TCGGGGCTCTTTGGGCTCTAAA |
| CTNBB1 | CACAAGCAGAGTGCTGAAGGTG | GATTCCTGAGAGTCCAAAGACAG |
| CMYC | CCTGGTGCTCCATGAGGAGAC | CAGACTCTGACCTTTTGCCAGG |

Development • Supplementary information

**Table S4.** List of western blot antibodies.

| Target | Dilution | Manufacturer | Cat Number | |
|---|---|---|---|---|
| ABC | 1:500 | Millipore | 05-665 | Primary |
| TBC | 1:1000 | Abcam | ab32572 | |
| AXIN2 | 1:500 | Origene | TA319745 | |
| Anti-rabbit | 1:10000 | LI-COR | Q92343-1 | Secondary |
| Anti-mouse | 1:10000 | LI-COR | Q92343-1 | |
| Anti-goat | 1:10000 | LI-COR | Q92343-1 | |

Development • Supplementary information
